# Palmitoylation of glycoproteins under low temperature enhances SVCV replication

**DOI:** 10.1371/journal.ppat.1014654

**Published:** 2026-09-28

**Authors:** Chen Li, Yan Zhang, Yujun Zhang, Yan Gao, Yuanan Lu, Shengbo Cao, Jing Ye, Xueqin Liu

**Affiliations:** 1 National Key Laboratory of Agricultural Microbiology, College of Fisheries, Huazhong Agricultural University, Wuhan, Hubei, China; 2 Hubei Engineering Technology Research Center for Aquatic Animal Diseases Control and Prevention, Wuhan, Hubei, China; 3 Hubei Jiangxia Laboratory, Wuhan, China; 4 Ocean College, Hebei Agricultural University, Qinhuangdao, Hebei, China; 5 Department of Public Health Sciences, the University of Hawaii at Manoa, Honolulu, Hawaii, United States of America; 6 National Key Laboratory of Agricultural Microbiology, College of Veterinary Medicine, Huazhong Agricultural University, Wuhan, Hubei, China; University of Tokyo Graduate School of Medicine Faculty of Medicine: Tokyo Daigaku Daigakuin Igakukei Kenkyuka Igakubu, JAPAN

## Abstract

The temperature of water is a crucial factor that affects rhabdovirus infections in fish. Numerous rhabdoviruses, known for their high infectivity at relative low water temperatures (below 20°C), have led to significant economic impacts on global aquaculture. However, the molecular mechanisms underlying their temperature-dependent infectivity and pathogenicity remain unclear. Here, we employed spring viremia of carp virus (SVCV), a rhabdovirus prevalent at low temperatures, as a model. We found that the low temperature markedly elevates levels of palmitic acid in zebrafish, which promotes the palmitoylation of the SVCV glycoprotein (G). This modification enhances stability and cell membrane localization of the G protein, thereby facilitating viral budding. Furthermore, we identified the acyltransferase ZDHHC15a, upregulated at low temperatures, as the mediator of this palmitoylation. A competitive peptide targeting the palmitoylation site of SVCV G exhibited potent antiviral activity. Notably, a similar mechanism was observed in other aquatic rhabdoviruses that are low-temperature susceptible. Overall, our findings elucidate the mechanism underlying the temperature sensitivity of fish rhabdoviruses and hold broader significance for understanding the temperature adaptive evolution of other aquatic viruses.

## Author summary

Environmental temperature critically influences pathogen–host interactions, but the underlying molecular adaptations remain unclear. Using SVCV-infected zebrafish, we show that low temperature activates fatty acid synthesis, driving palmitoylation of the viral G protein via the transferase ZDHHC15a. This modification stabilizes G, enhances membrane localization, and promotes budding—a mechanism conserved in low temperature-adapted rhabdoviruses. A rationally designed peptide blocking this palmitoylation causes G degradation and suppresses replication. Our findings reveal fatty-acid-mediated palmitoylation as a temperature-sensitive switch for viral infectivity, offering evolutionary and therapeutic insights.

## Introduction

In aquatic ectotherms, exposure to thermal stress either cold or heat profoundly affects growth, is closely associated with the occurrence of viral diseases. Consequently, viral disease outbreaks in aquaculture typically exhibit strong seasonality and temperature dependence [1,2]. Among these, rhabdovirus infections in fish have caused substantial global economics losses and are particularly sensitive to temperature fluctuations. Outbreaks of viral hemorrhagic septicemia virus (VHSV), infectious hematopoietic necrosis virus (IHNV), hirame rhabdovirus (HIRRV), and spring viremia of carp virus (SVCV) primarily occur at water temperatures below 20°C [3–6]. In contrast, Siniperca chuatsi rhabdovirus (SCRV) and Micropterus salmoides rhabdovirus (MSRV) predominate in warm waters, with optimal replication between 25°C to 28°C [7,8]. The low-temperature-susceptible viruses, VHSV, IHNV, and HIRRV belong to the genus *Novirhabdovirus*, whose genome encodes six structural proteins in the order: nucleoprotein (N), phosphoprotein (P), matrix protein (M), glycoprotein (G), non-virion protein (NV), and RNA-dependent RNA polymerase (L) [9–11]. Another low-temperature-prevalent virus, SVCV, classified within the genus *Sprivivirus*, lacks the NV gene [6]. Elucidating the molecular mechanisms underlying this pronounced temperature-dependent prevalence in aquatic rhabdoviruses is crucial for developing targeted prevention and control strategies in aquaculture.

Virus-host interactions constitute a continuous co-evolutionary process in which hosts develop defense mechanism to restrict viral infections, whereas viruses evolve counterstrategies to maintain persistence and replication [9]. As obligate intracellular parasites, viruses extensively exploit host cellular machinery - particularly metabolic pathway - to complete their life cycle [10]. Classical host metabolic networks – though essential for maintaining host homeostasis and defense, are frequently hijacked by viruses to support replication and assembly [11–16]. Among these pathways, lipid metabolism plays a pivotal role, influencing cellular energy balance, membrane dynamics, endocytosis, signaling cascades, and viral assembly or budding [17,18]. Notably, the fatty acid synthesis pathway is an evolutionarily conserved metabolic axis crucial for host adaptation to both temperature stress and viral infection [10,19–22]. Despite these insights, the mechanistic links between temperature, fatty acid metabolism, and viral infection remain poorly understood.

Alternations in lipid metabolism can lead to changes in specific classes of intracellular lipid modifications [23]. Based on the three major lipid groups - fatty acids, isoprenoids, and glycosylphosphatidylinositol (GPI) - lipid modifications are generally categorized into acylation, isoprenylation, and GPI anchoring. Lipid acylation primarily includes myristoylation (C14:0, N-myristoylation) and palmitoylation (C16:0, S-palmitoylation) [24]. Palmitoylation in protein usually means that palmitic acid is connected to cysteine through thioester bond, so it is also called S-palmitoylation. In addition to palmitic acid (C16:0), other long-chain fatty acyl-CoAs such as palmitoleic acid (C16:1), stearic acid (C18:0), and oleic acid (C18:1) can also serve as substrates for protein S-palmitoylation [25]. Functionally, palmitoylation increases the hydrophobicity of viral transmembrane proteins, regulates their intracellular trafficking and membrane localization, and thereby modulates membrane fusion, viral assembly, and release during infection [26,27].

In this study, we employed SVCV infection in zebrafish as a homologous virus-host model to elucidate the molecular mechanisms governing the temperature-dependent behavior of rhabdoviruses in fish. We found that the fatty acid synthesis pathway is activated at low temperature. which facilitates palmitoylation of the viral glycoprotein (G) mediated by palmitoyl transferase ZDHHC15a. This modification stabilizes the G protein and enhances its membrane localization, thereby promoting viral budding. Notably, this mechanism appears conserved among other low-temperature-susceptible fish rhabdoviruses. Treatment of a short peptide that inhibits this palmitoylation leads to intracellular degradation of the G protein and suppression of viral replication. Collectively, this study provides the first comprehensive demonstration that fatty acid-mediated palmitoylation of viral proteins is a critical determinant of temperature-dependent pathogenesis of aquatic rhabdoviruses.

## Results

### The temperature dependence of SVCV infection is related to fatty acid synthesis

To assess the relationship between the infectivity of aquatic rhabdovirus and water temperature, three aquatic rhabdoviruses, including SVCV, IHNV, and VHSV, which are known to be susceptible at low temperatures, were employed. Zebrafish were infected with SVCV at 16 °C and 28 °C, while rainbow trout were infected with IHNV or VHSV at 15 °C and 25 °C. As expected, the results indicate that zebrafish or rainbow trout exhibit higher susceptibility to these viruses at the lower temperature (S1A-S1C Fig). Subsequently, Zebrafish embryo fibroblast-like (ZF4) cells were infected with SVCV, IHNV, and VHSV respectively at both 16 °C and 28 °C. All three viruses demonstrated significantly higher intracellular genomic RNA levels (S1D-S1F Fig) and extracellular infectious viral titers (S1G-S1I Fig) at 16 °C compared to those at 28 °C. These findings confirm that temperature is a critical factor influencing the progression of aquatic *rhabdovirus* infections.

To further elucidate the mechanism underlying the temperature dependence of aquatic rhabdovirus infectivity and pathogenicity, we conducted transcriptomic analysis with the spleen tissues of SVCV-infected zebrafish at 16 °C and 28 °C. The results suggest that the fatty acid metabolic pathways, particularly the FASN-mediated pathway involved in fatty acid synthesis, were remarkably activated at the low temperature (16 °C) (S2A-S2B Fig). In addition, we analyzed the metabolomics data of zebrafish tissues at different temperatures (16 °C and 28 °C) that were previously reported by our laboratory [28]. KEGG pathway analysis, following the exclusion of non-lipid metabolites, revealed that low temperature significantly upregulated the fatty acid biosynthesis pathway, leading to a marked increase in the production of fatty acids, including palmitic acid and stearic acid (S2C-S2D Fig). Further gas chromatography and qPCR results confirmed a significant increase in palmitic acid content in ZF4 cells (S2E Fig) and a higher expression level of fatty acid synthesis enzyme genes in zebrafish tissues (Fig 1A) at 16 °C. These findings suggest that the fatty acid synthesis pathway is activated at low temperatures.

**Fig 1:**
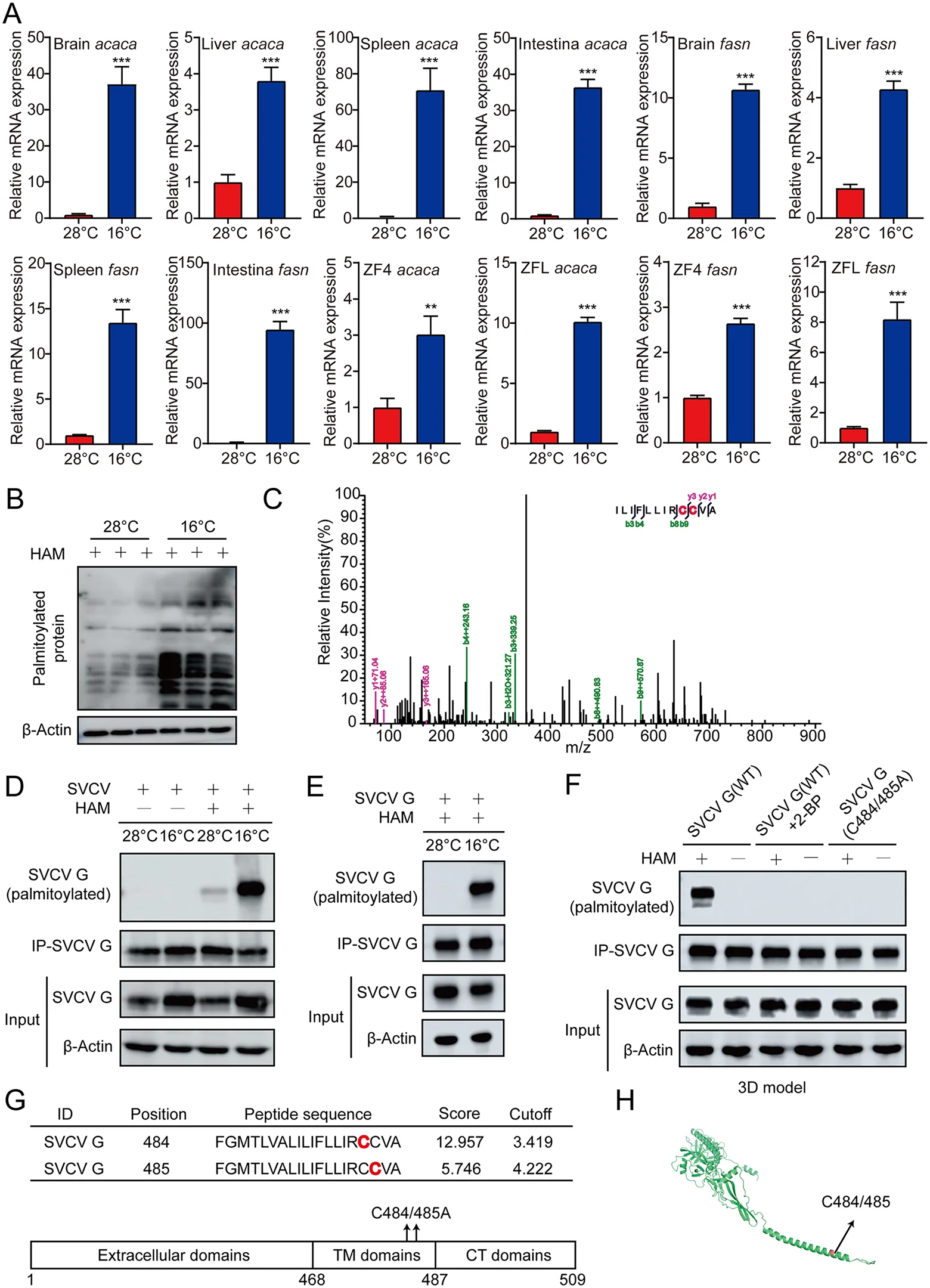
Palmitoylation of the SVCV G protein contributes to the increased viral proliferation at low temperatures. **(A)** The expression of *acaca* and *fasn* in the brain, liver, spleen, intestine of zebrafish from different temperature were quantified by qRT-PCR. The expression of *acaca* and *fasn* in ZF4 cells from different temperatures were quantified by qRT-PCR. **(B)** The palmitoylation levels of proteins in ZF4 cells were analyzed at different temperatures. **(C)** Palmitoylated peptides were found in SVCV G protein by palmitoylated mass spectrometry. **(D)** ZF4 cells were infected with SVCV (MOI = 10) for 24 h at 16 °C and 28 °C. The palmitoylation level of SVCV G was measured by IP-ABE assay, and then cells were blotted with streptavidin-HRP and SVCV G mAb. -HAM, without HAM; + HAM, with HAM. **(E)** ZF4 cells were transfected with plasmids expressing SVCV G at 16 °C and 28 °C. Protein palmitoylation was examined by IP-ABE assay in the presence of HAM. The palmitoylated SVCV G and the immunoprecipitated SVCV G were blotted by HRP-streptavidin and SVCV G mAb, respectively. **(F)** ZF4 cells were transfected with plasmids expressing SVCV G/WT and SVCV G/C484/485A at 16 °C and subsequently mock treated (SVCV G/WT and SVCV G/C484/485A groups) or treated with 50 mM 2-BP (SVCV G/WT 2-BP group). Protein palmitoylation was examined by IP-ABE assay in the presence and absence of HAM. The palmitoylated SVCV G and the immunoprecipitated SVCV G were blotted by HRP-streptavidin and SVCV G mAb, respectively. **(G)** Protein palmitoylation software CSS-palm 4.0 was used to predict the SVCV G palmitoylation sites. **(H)** 3D model of the palmitoylation site of SVCV G protein. The predicted structure of SVCV G from the AlphaFold Protein Structure Database (AFDB) using its UniProt accession number Q88415. The structure corresponds to the AlphaFold entry AF-0000000365761399, with a predicted Local Distance Difference Test (pLDDT) score of 73.95. Data was presented as mean ± SD from three independent experiments. Statistical analysis was performed using Student’s *t*-test between different groups and the asterisk (*) indicates significant differences between groups*p < 0.05, **p < 0.01, ***p < 0.001.

To investigate the involvement of the fatty acid synthesis pathway in SVCV infection, the low temperature (16 °C) adapted ZF4 cells were treated with an Acetyl-CoA Carboxylase alpha (ACACA) inhibitor, Firsocostat, or a fatty acid synthase (FASN) inhibitor, Orlistat, at a nontoxic concentration followed by SVCV infection. We found that Firsocostat and Orlistat treatment both significantly inhibited the production of SVCV RNA, proteins, infectious virions at 16 °C (S3A-S3B Fig, S3D-S3I Fig). These findings imply that fatty acid synthesis plays a crucial role in the process of SVCV infection at low temperatures.

### Palmitoylation of the SVCV G protein facilitates viral proliferation at low temperatures

Palmitic acid, which is remarkably upregulated in SVCV-infected zebrafish at low temperature, can be directly utilized for palmitoylation (S-acylation) at cysteine residues of target proteins. Therefore, we subsequently assessed whether the activation of FASN pathway at low temperatures affects the palmitoylation modification process. The results of the acyl-biotin exchange (ABE) assay revealed a significant increase in the palmitoylation level of total proteins in ZF4 cells cultured at 16 °C compared to those cultured at 28 °C following SVCV infection (Fig 1B). To identify target proteins exhibiting differential palmitoylation, we conducted palmitoylation LC-MS analysis on ZF4 cells infected with SVCV under different temperatures (16 °C and 28 °C). Notably, the SVCV G protein was identified to exhibit significant palmitoylation modification at the low temperature (16 °C). A secondary mass spectrometry analysis revealed that cysteine (C) residues 484 and 485 of the SVCV G protein were palmitoylated (Fig 1C). The LC-MS results were further verified by the IP-ABE assay (Fig 1D) We next compared the palmitoylation modification levels of the exogenously expressed SVCV G protein at different temperatures with the ZF4 cells transfected with a plasmid encoding SVCV G. The results revealed a significantly higher level of palmitoylation in the SVCV G protein at 16 °C compared to 28 °C (Fig 1E). Additionally, treatment of ZF4 cells expressing SVCV G with the palmitoylation inhibitor 2-bromopalmitate (2-BP) resulted in the absence of bands corresponding to palmitoylated SVCV G protein, as demonstrated by the IP-ABE assay provided conclusive evidence that SVCV G is palmitoylated at low temperature (Fig 1F). We further employed the palmitoylation analysis software CSS-Palm 4.0, which predicted the consistent palmitoylated sites, C484 and C485, in SVCV G protein and no palmitoylation modification sites in SVCV N, P, M, and L proteins (Fig 1G). Notably, the predicted sites for palmitoylation modification of SVCV G protein are located within its transmembrane region (Fig 1G-1H). To validate this prediction, C484 and C485 in SVCV G protein were substituted with serine (C484/485A). Subsequently, ZF4 cells expressing SVCV G WT protein or C484/485A mutant were subjected to IP-ABE assays. The results revealed the absence of palmitoylation of SVCV G C484/485A mutant (Fig 1F). Collectively, these findings suggest that SVCV G undergoes palmitoylation modification, with C484 and C485 serving as the major palmitoylation sites.

To clarify whether palmitoylation of the SVCV G protein is associated with enhanced viral proliferation at low temperatures, ZF4 cells cultured at 16 °C were treated with increasing concentrations of 2-BP (10, 25, 50, and 100 μM), followed by infection with SVCV. After 24 h of viral infection, we observed a dose-dependent reduction in viral RNA and protein levels, as well as infectious viral titers, in the 2-BP-treated cells, compared to the mock-treated controls (S3C Fig, S3J-S3L Fig), suggesting that the palmitoylation of SVCV G protein contributes to increased viral production at low temperatures.

To investigate at which stage of the viral life cycle palmitoylation affects SVCV infection, we examined the effects of 2-BP on early viral entry and viral replication. The results showed that palmitoylation does not affect early SVCV entry and viral replication (S3M-S3N Fig).

### Palmitoylation of the SVCV G protein is essential for viral budding

Given the pivotal role of palmitoylation in modulating protein stability, we examined its impact on the stability of SVCV G protein. ZF4 cells, ectopically expressing SVCV G, were cultured at 16°C and 28 °C, and the stability of the G protein was assessed following treatment of Cycloheximide (CHX), a translation inhibitor. The SVCV G protein exhibited accelerated degradation at 28 °C compared to 16°C (Fig 2A-2B), suggesting enhanced protein stability under low-temperature conditions. In addition, compared to the WT SVCV G protein, the palmitoylation-deficient SVCV G C484/485A mutant expressed at 16°C exhibited an enhanced degradation (Fig 2C). Furthermore, we found that both Orlistat and 2-BP treatments induced a dose-dependent degradation of the ectopically expressed SVCV G protein (S4A Fig and Fig 2D). Collectively, these findings suggest that the palmitoylation of the SVCV G protein enhances its stability.

**Fig 2:**
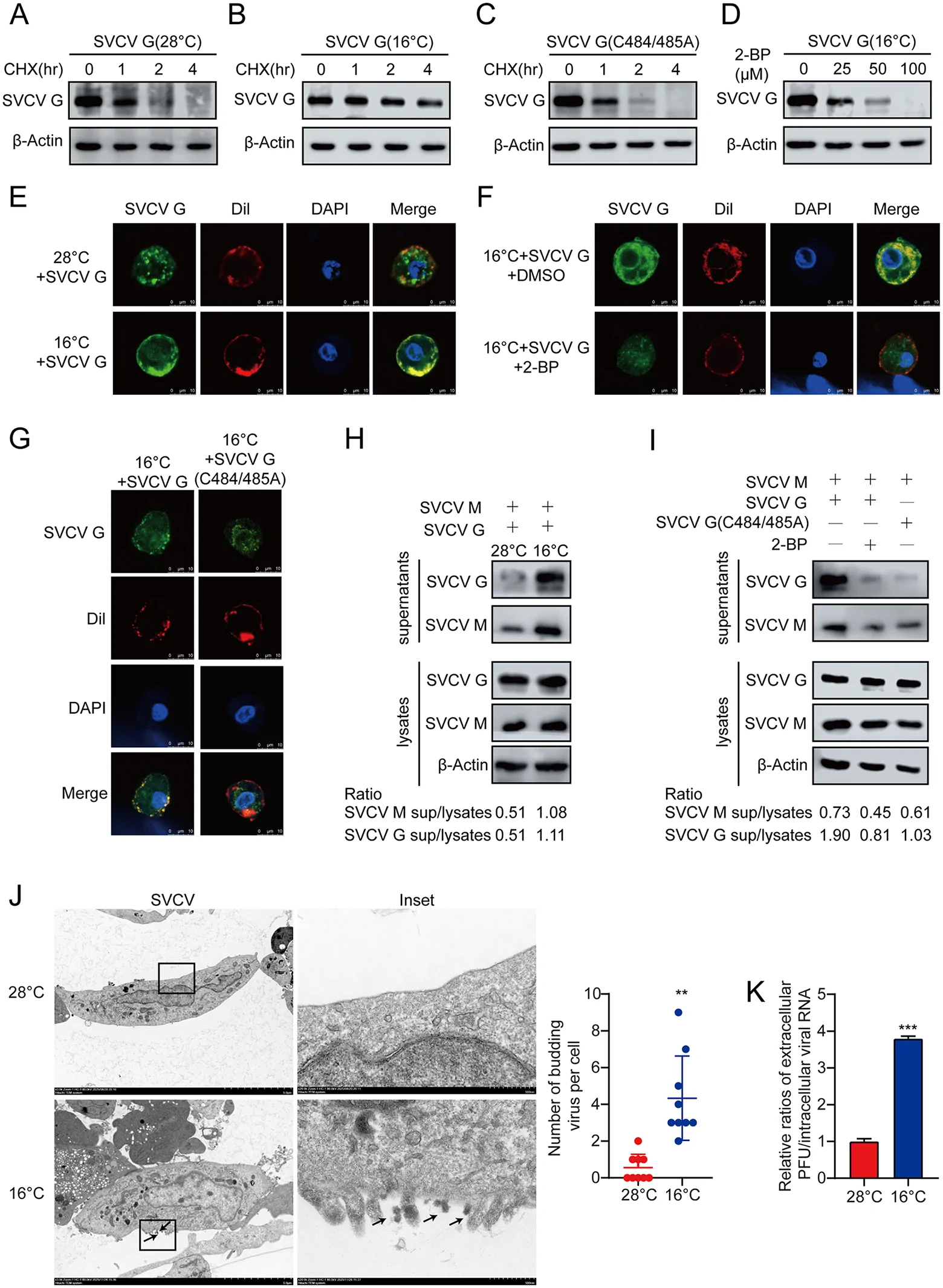
Palmitoylation of the SVCV G protein is essential for viral budding. **(A-B)** ZF4 cells were transfected with the plasmid encoding SVCV G for 24h at 28 °C and 16 °C. Then, the cells were stimulated with CHX (1 µg/mL) and lysed at the indicated time points (0 h, 1 h, 2 h, and 4 **h)**. The protein levels of SVCV-G in the cell lysate were measured by Western blotting. **(C)** ZF4 cells were transfected with the plasmid encoding SVCV G(C484/485A) for 24h at 28 °C and 16 °C. Then, the cells were stimulated with CHX (1 µg/mL) and lysed at the indicated time points (0 h, 1 h, 2 h, and 4 **h)**. The protein level of SVCV-G in the cell lysate was measured by Western blotting. **(D)** ZF4 cells were transfected with the plasmid encoding SVCV G and were then treated with 2-BP (25µM, 50 µM, and 100 µM) or DMSO for 24 h at 16 °C. The SVCV G protein levels were measured by Western blotting. **(E)** ZF4 cells were transfected with EGFP-SVCV G plasmid for 24 h at 16°C and 28°C. Cell membranes were stained with Dil and nuclei with DAPI. Scale bars,10 μm. **(F)** ZF4 cells expressing EGFP-SVCV G were treated with control solvent or 2-BP (50 μM) for 24 h at 16°C. Cell membranes were stained with Dil and nuclei with DAPI. Scale bars,10 μm. **(G)** ZF4 cells were transfected with EGFP-SVCV G or the mutants for 24 h at 16°C. Cell membranes were stained with Dil and nuclei with DAPI. Scale bars,10 μm. **(H)** ZF4 cells were co-transfected with SVCV G and SVCV M for 24 h at 16°C and 28 °C. The protein levels of SVCV-M and SVCV-G in the cell lysate and culture supernatants were measured by Western blotting. **(I)** ZF4 cells were transfected with plasmids expressing SVCV G/the mutants and SVCV M at 16 °C and subsequently mock treated (SVCV G/the mutants and SVCV M groups) or treated with 50 mM 2-BP (SVCV G 2-BP group). The protein levels of SVCV-M and SVCV-G in the cell lysate and culture supernatants were measured by Western blotting. **(J)** ZF4 cells were infected with SVCV (MOI = 10) under different temperature conditions. Following 12 hours post-infection, the cells were fixed with glutaraldehyde and processed for transmission electron microscopy to observe SVCV budding. Representative images are shown in (J), with scale bars of 5 μm and 1 μm. **(K)** The budding efficiency of SVCV in ZF4 cells cultured at different temperatures was assessed by calculating the ratio of intracellular and extracellular infectious virus titers to the total infectious titer at 12 hours post-infection. Data was presented as mean ± SD from three independent experiments. Statistical analysis was performed using Student’s *t*-test between different groups and the asterisk (*) indicates significant differences between groups*p < 0.05, **p < 0.01, ***p < 0.001.

To investigate the specific pathway through which palmitoylation affects the stability of SVCV G protein, ZF4 cells expressing SVCV G were initially treated with 2-BP, followed by addition of the ubiquitin-proteasome pathway inhibitor MG132, the autophagy inhibitor 3-MA, or the lysosomal pathway inhibitor NH_4_Cl or Baf-A1. The results revealed that treatment with MG132 and 3-MA didn’t affect the levels of SVCV G protein (S4B-S4C Fig), whereas treatment with NH_4_Cl and Baf-A1 significantly increased the abundance of SVCV G protein (S4D-S4E Fig), compared to mock-treated controls. These findings indicate that the degradation of SVCV G protein induced by 2-BP primarily occurs via the lysosomal pathway. To further clarify whether the removal of palmitoylation leads to the degradation of SVCV G protein via the lysosomal pathway, we isolated lysosomes from ZF4 cells treated or untreated with 2-BP following transfection with the SVCV G construct to quantify the abundance of the G protein within the lysosomes. Our results showed a higher level of SVCV G protein in lysosomes at 28 °C dthan those at 16 °C (S4F Fig). Additionally, lysosomes from ZF4 cells treated with 2-BP exhibited a significantly higher abundance of SVCV G protein compared to the vehicle-treated group (S4G Fig). Furthermore, the abundance of palmitoylation-deficient SVCV G protein in lysosomes was also significantly higher than that of WT SVCV G protein (S4H Fig). These results suggest that palmitoylation of the SVCV G protein at low temperatures prevents its lysosomal degradation.

As the stability of the SVCV G protein expressed in host cells may influence viral assembly and budding from the cell membrane, we further assessed the cellular localization of SVCV G protein at different temperatures. Interestingly, the SVCV G protein was predominantly localized on the cell membrane at 16 °C, while a substantial cytoplasmic localization of this protein was observed at 28 °C (Fig 2E, S4I Fig). 2-BP treatment disrupted the localization of the SVCV G protein to the cell membrane at 16 °C (Fig 2F, S4J Fig). Similarly, the SVCV G C484/485S mutant also failed to localize to the cell membrane (Fig 2G, S4K Fig). These results indicate that palmitoylation of the SVCV G protein is essential for its localization to the cell membrane.

To further investigate the efficiency of viral budding at different temperatures, ZF4 cells were co-transfected with plasmids encoding the SVCV M and G proteins to generate virus-like particles (VLPs) (S4L Fig), and the abundances of these proteins in both culture supernatants and cell lysates were quantified. The results revealed a marked increase in the ratios of SVCV M and SVCV G protein levels in the supernatant to those in the cell lysates at 16 °C (Fig 2H). This suggests an enhanced budding of SVCV VLPs at lower temperatures compared to that at higher temperatures. However, following treatment with 2-BP, the ratios of SVCV M and G protein levels in the supernatants versus those in the cell lysates were significantly reduced (Fig 2I). Furthermore, transfection of plasmid harboring mutations at the palmitoylation modification sites of SVCV G, along with SVCV M construct, resulted in a reduction in the ratios of both SVCV M and G proteins in the supernatant related to those in the cell lysate (Fig 2I).

To further verify the impact of temperature on the viral budding during SVCV infection, transmission electron microscopy was employed to visualize the progeny virions. A greater number of viral particles undergoing budding and being released from infected cells was observed at 16 °C compared to those infected at 28 °C (Fig 2J). Furthermore, analysis of the ratio of extracellular infectious viral particle numbers to intracellular viral RNA levels at different temperatures demonstrated a significantly higher proportion of released mature viral particles at 16 °C (Fig 2K). These findings collectively suggest that the palmitoylation of the SVCV G protein, which is enhanced under low-temperature conditions, facilitates the proper localization of the SVCV G protein on the cell membrane. This, in turn, enhances viral proliferation by promoting the budding of SVCV.

### ZDHHC15a mediates palmitoylation of SVCV G protein at low temperatures

To identify the specific palmitoyl transferase responsible for regulating the palmitoylation of the SVCV G protein, a comprehensive analysis of the transcriptomic data of zebrafish spleen across various temperature conditions was conducted. ZDHHC15a, a palmitoyl transferase belonging to the ZDHHC family, was identified to be significantly upregulated at 16 °C (S5A Fig). This upregulation was further confirmed using qRT-PCR and Western blot assays with zebrafish tissues (including brain, liver, spleen, and intestine) and cells (ZF4 and ZFL cells) across various temperatures (S5B-S5D Fig). Subsequently, ZDHHC15a was knocked down in ZF4 cells using specific siRNA followed by transfection with a plasmid encoding SVCV G. The IP-ABE assay demonstrated that the knockdown of ZDHHC15a significantly reduced both the palmitoylation level and the protein level of SVCV G (Fig 3A-3C). Furthermore, Co-IP assays demonstrated the interaction between both exogenous and endogenous ZDHHC15a and the SVCV G protein (S5E Fig and Fig 3D). The immunofluorescence assay further revealed that endogenous ZDHHC15a specifically colocalized with the SVCV G protein in zebrafish cells (S5F Fig). To further evaluate the role of ZDHHC15a in SVCV proliferation, ZF4 cells were transfected with siRNA targeting ZDHHC15a, followed by SVCV infection at 16 °C. The knockdown of ZDHHC15a significantly suppressed the proliferation of SVCV under low-temperature conditions (Fig 3E-3F). These data suggest that ZDHHC15a, which is induced by low temperatures, functions as a palmitoyl transferase for the SVCV G protein and facilitates SVCV infection at low temperatures.

**Fig 3:**
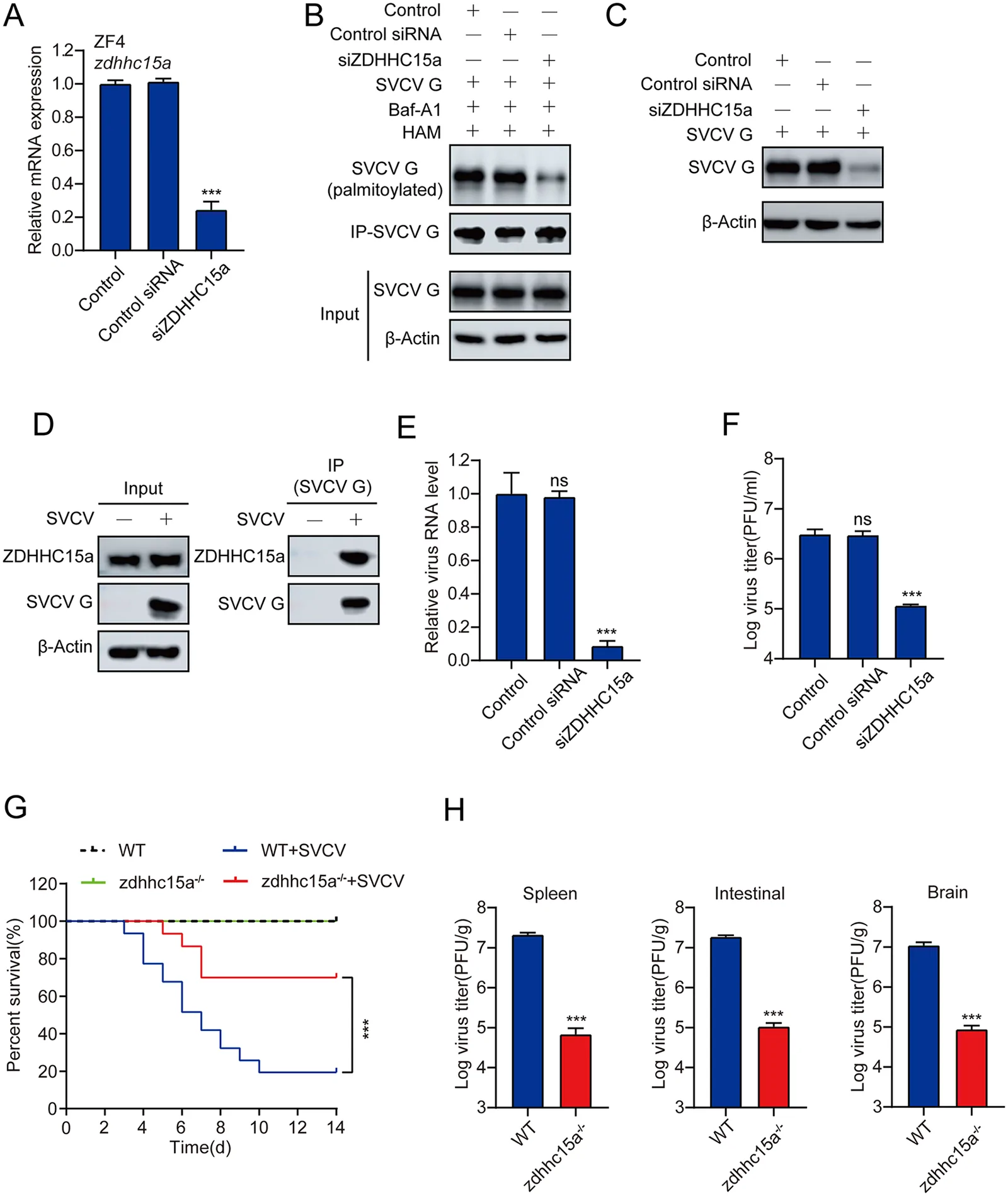
The low temperature-induced acyltransferase ZDHHC15a mediates palmitoylation of SVCVG. **(A)** RNA interference effect on the expression of endogenous ZDHHC15a. ZF4 cells were transfected with ZDHHC15a-specific siRNA or control siRNA (NC) for 24 **h.** The efficiency of siRNAs to inhibit the ZDHHC15a expression was detected by qRT-PCR. **(B)** ZF4 cells were transfected with si-ZDHHC15a or si-NC for 12 h and then were transfected with the plasmid encoding SVCV G for 24 **h.** Protein palmitoylation was examined by IP-ABE assay. The palmitoylated SVCV G and the immunoprecipitated SVCV G were blotted by HRP-streptavidin and SVCV G mAb, respectively. **(C)** ZF4 cells were transfected with si-ZDHHC15a or si-NC for 12 h and then were transfected with the plasmid encoding SVCV G for 24 **h.** Then, the cells were analyzed for SVCV G expression. **(D)** ZF4 cells were infected with SVCV for 24 **h.** The supernatant of WCL was immunoprecipitated using anti-SVCV G magnetic beads magnetic beads. Then, the precipitated proteins were analyzed by Western blotting. **(E-F)** ZF4 cells were transfected with si-ZDHHC15a or si-NC for 12 h and then infected with SVCV(MOI = 10) for 24 **h.** Cell samples and supernatant were collected 24 h after infection. The viral RNA abundance was detected using qRT-PCR. SVCV titers were measured by Viral plaque assay. **(G)** Percentage survival of wild-type and *zdhhc15a*^-/-^ zebrafish (n = 30) injected with 10 µl SVCV (10^7^PFU/ml). Statistics of survival rate were calculated with the log-rank (Mantel-Cox). ***p < 0.001. **(H)** The virus titer in tissues were detected by virus titration in zebrafish. Data was presented as mean ± SD from three independent experiments. Statistical analysis was performed using Student’s *t*-*t*est between different groups and the asterisk (*) indicates significant differences between groups*p < 0.05, **p < 0.01, ***p < 0.001.

To verify the biological role of ZDHHC15a in SVCV infection *in vivo*, the *zdhhc15a* gene knockout (KO) zebrafish were generated using CRISPR-Cas9 technology (S5G-S5H Fig). WT and *zdhhc15a* KO zebrafish were infected with SVCV at 16 °C. Compared to the infected-WT zebrafish, the markedly attenuated symptoms, such as skin hemorrhaging and ascites, as well as enhanced survival rate were observed in *zdhhc15a* KO zebrafish following SVCV infection (S5I Fig and Fig 3G). In addition, zdhhc15a deficiency also significantly reduced the viral load in various tissues (Fig 3H). These results further confirmed the important role of ZDHHC15a in SVCV infection *in vivo*.

### G protein palmitoylation is correlated with the temperature-dependent infectivity of *rhabdoviruses*

To investigate the relationship between the palmitoylation-regulated stability of G protein and the temperature-sensitivity of different rhabdoviruses, we selected two other viruses that exhibit high infectivity at low temperatures: VHSV (optimal infection temperature range: 6–12 °C) and IHNV (optimal infection temperature range: 8–12 °C), as well as MSRV and SCRV, which are susceptible at high temperatures (optimal infection temperature range: 25–28 °C), were selected to predict the palmitoylation modification sites in their G proteins. The prediction results reveal that both VHSV and IHNV possess palmitoylation modification sites, with VHSV exhibiting a greater number of palmitoylation sites in its G protein, correlating with its optimal proliferation at lower temperatures compared to IHNV. In contrast, no palmitoylation modification sites were predicted in MSRV and SCRV G proteins (Fig 4A). Consistently, IP-ABE analysis identified an evident palmitoylation modification in the VHSV and IHNV G proteins; however, no such modification was observed in both G proteins harboring palmitoylation site mutations (Fig 4B-4C). Additionally, the loss of palmitoylation in the VHSV and IHNV G proteins was confirmed following treatment with 2-BP (Fig 4B-4C). Furthermore, the palmitoylation levels of both VHSV and IHNV G proteins were significantly elevated at the lower temperature compared to the higher temperature (Fig 4D-4E). In contrast, IP-ABE assay confirmed that the G protein of MSRV does not exhibit palmitoylation modification (Fig 4F). These results suggest that palmitoylation modifications of the G proteins broadly occur in low-temperature susceptible *rhabdoviruses,* but not in those that thrive at higher temperatures.

**Fig 4:**
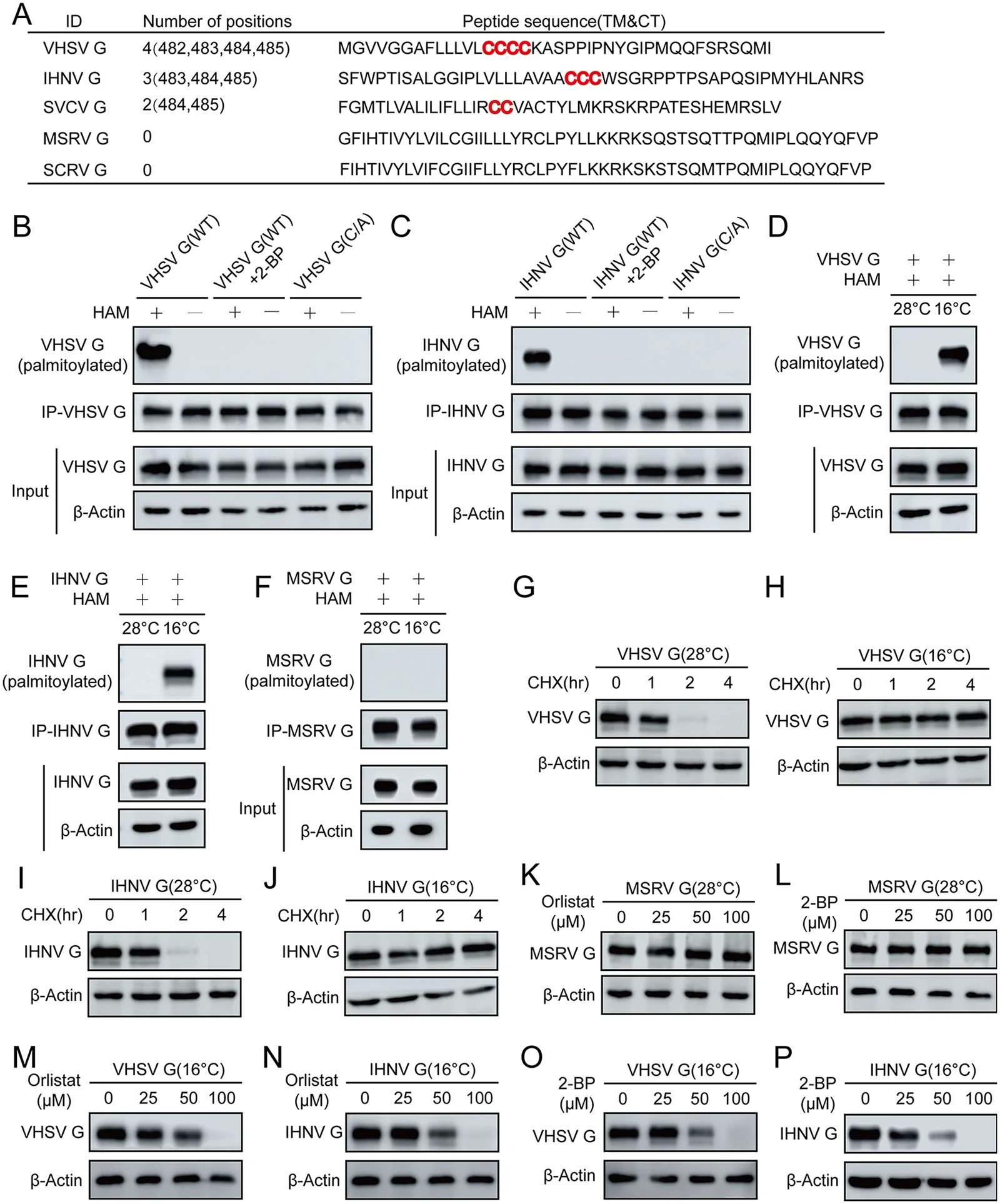
The G protein of low-temperature-sensitive *rhabdovirus* is palmitoylated. **(A)** Protein palmitoylation software CSS-palm 4.0 was used to predict the VHSV G, IHNV G, SVCV G, MSRV G palmitoylation sites. **(B)** ZF4 cells were transfected with plasmids expressing VHSV G/WT and VHSV G(C/A) at 16 °C and subsequently mock treated (VHSV G/WT and VHSV G(C/A) groups) or treated with 50 μM 2-BP (VHSV G/WT 2-BP group). Protein palmitoylation was examined by IP-ABE assay in the presence and absence of HAM. The palmitoylated VHSV G and the immunoprecipitated VHSV G were blotted by HRP-streptavidin and His mAb, respectively. **(C)** ZF4 cells were transfected with plasmids expressing IHNV G/WT and IHNV G(C/A) at 16 °C and subsequently mock treated (IHNV G/WT and IHNV G(C/A) groups) or treated with 50 μM 2-BP (IHNV G/WT 2-BP group). Protein palmitoylation was examined by IP-ABE assay in the presence and absence of HAM. The palmitoylated IHNV G and the immunoprecipitated IHNV G were blotted by HRP-streptavidin and His mAb, respectively. **(D-F)** ZF4 cells were transfected with plasmids expressing VHSV G, IHNV G and MSRV G at 16 °C and 28 °C. Protein palmitoylation was examined by IP-ABE assay in the presence of HAM. The palmitoylated MSRV G, VHSV G and IHNV G and the immunoprecipitated VHSV G, IHNV G and MSRV G were blotted by HRP-streptavidin and His mAb, respectively. **(G-H)** ZF4 cells were transfected with the plasmid encoding VHSV G for 24h at 28 °C and 16 °C. Then, the cells were stimulated with CHX (1 µg/mL) and lysed at the indicated time points (0 h, 1 h, 2 h, and 4 **h)**. The protein levels of VHSV G in the cell lysate were measured by Western blotting. **(I-J)** ZF4 cells were transfected with the plasmid encoding IHNV G for 24h at 28 °C and 16 °C. Then, the cells were stimulated with CHX (1 µg/mL) and lysed at the indicated time points (0 h, 1 h, 2 h, and 4 **h)**. The protein levels of IHNV G in the cell lysate were measured by Western blotting. **(K)** ZF4 cells were transfected with the plasmid encoding MSRV G and were then treated with Orlistat (25µM, 50 µM, and 100 µM) or DMSO for 24 h at 16 °C. The MSRV G protein levels were measured by Western blotting. **(L)** ZF4 cells were transfected with the plasmid encoding MSRV G and were then treated with 2-BP (25µM, 50 µM, and 100 µM) or DMSO for 24 h at 16 °C. The MSRV G protein levels were measured by Western blotting. **(M-N)** ZF4 cells were transfected with the plasmid encoding VHSV G and IHNV G and were then treated with Orlistat (25µM, 50 µM, and 100 µM) or DMSO for 24 h at 16 °C. The VHSV G and IHNV G protein levels were measured by Western blotting. **(O-P)** ZF4 cells were transfected with the plasmid encoding VHSV G and IHNV G and were then treated with 2-BP (25µM, 50 µM, and 100 µM) or DMSO for 24 h at 16 °C. The VHSV G and IHNV G protein levels were measured by Western blotting.

We subsequently evaluated the thermal stability of the VHSV and IHNV G proteins. Our observation indicated that these proteins exhibited enhanced stability when expressed at lower temperatures (Fig 4G-4J). However, 2-BP treatment markedly reduced the stability of both VHSV and IHNV G proteins at 16 °C (Fig 4M-4P), while having no effect on the levels of MSRV G protein (Fig 4K-4L). Furthermore, we investigated the effect of Orlistat and 2-BP on proliferation of VHSV and IHNV. Both inhibitors significantly inhibited the production of RNA levels and infectious virions of both VHSV and IHNV (S6A-S6H Fig). These findings suggest that palmitoylation bolsters the stability of the G protein and facilitates proliferations of *rhabdoviruses* that are highly infectious at low temperatures.

Next, we evaluated the role of ZDHHC15a in palmitoylation of the VHSV and IHNV G proteins. Co-IP analysis demonstrated that exogenous ZDHHC15a interacts with both VHSV and IHNV G proteins (S6I-S6J Fig). IP-ABE assay demonstrated a considerable decrease in the palmitoylation levels of both VHSV and IHNV G proteins upon ZDHHC15a knockdown (S6K-S6J Fig). Notably, ZDHHC15a knockdown resulted in significantly reduced viral RNA levels and titers (S6M-S6P Fig). These findings demonstrate that ZDHHC15a contributes to the palmitoylation of VHSV and IHNV G proteins and facilitates viral proliferation at low temperatures, revealing a conserved mechanism utilized by various low temperature-susceptible *rhabdoviruses.*

### Targeting the palmitoylation of G protein provides therapeutic effect on infections of low temperature-adaptive *rhabdoviruses*

To evaluate whether the palmitoylated G protein can serve as an antiviral target for low-temperature susceptible *rhabdoviruses*, we designed a short peptide consisting of the identified palmitoylation motif of SVCV G, which may act as a substrate for ZDHHC15a, thereby competitively inhibiting the palmitoylation of SVCV G. Transfecting ZF4 cells with a plasmid expressing GFP fused with the peptide containing the palmitoylation motif of SVCV G (GFP-GS1) at 16 °C revealed that the fused protein localized to the cell membrane (S7A Fig). In contrast, the GFP protein fused with the peptide containing the mutated palmitoylation motifs (GFP-GS2) was predominantly localized to the cytoplasm (S7A Fig). Consistently, the IP-ABE assays demonstrated significant palmitoylation of GFP-GS1, whereas no palmitoylation was observed for GFP-GS2 (S7B Fig). These findings indicate that this peptide exhibit characteristics consistent with the palmitoylated SVCV G protein. Notably, the expression of GFP-GS1, but not GFP-GS2, significantly inhibited both the palmitoylation and the protein level of exogenously expressed SVCV G (S7C-S7D Fig), as well as SVCV proliferation (S7E-S7G Fig). To determine whether GS1 interacts with ZDHHC15a as a substrate, we co-transfected GFP-GS1 or GFP-GS2 with the ZDHHC15a-Flag eukaryotic expression plasmid. Subsequent Co-IP experiments revealed an interaction between GS1 and ZDHHC15a (S7H Fig). To further determine whether GS1, as a substrate of ZDHHC15a, influences the interaction between ZDHHC15a and SVCV G, we treated cells co-transfected with eukaryotic expression plasmids encoding ZDHHC15a and SVCV G with CPP-GS1 and CPP-GS2, respectively. Subsequent Co-IP analysis revealed that CPP-GS1 significantly inhibited the interaction between ZDHHC15a and SVCV G (S7I Fig). These findings suggest that the peptide mimicking the palmitoylation motif of the SVCV G protein can competitively inhibit the palmitoylation of SVCV G, thereby suppressing viral proliferation.

To facilitate the penetration of peptide into the host cells, we next synthesized fusion peptides by combining cell-penetrating peptide (CPP) with GS1 and GS2 (Fig 5A). We measured the penetration efficiency of FAM-labeled CPPtat-GS1 in ZF4 cells, and the results showed that CPPtat-GS1 has a high ability to enter cells (S7J Fig). These peptides were subsequently added into the supernatant of ZF4 cells expressing SVCV G. Cells treated with CPP-GS1 exhibited a significant reduction in both the palmitoylation and the protein levels of SVCV G protein (Fig 5B-5C) and led to a significant suppression of SVCV proliferation in a dose-dependent manner (Fig 5D-5G).

**Fig 5:**
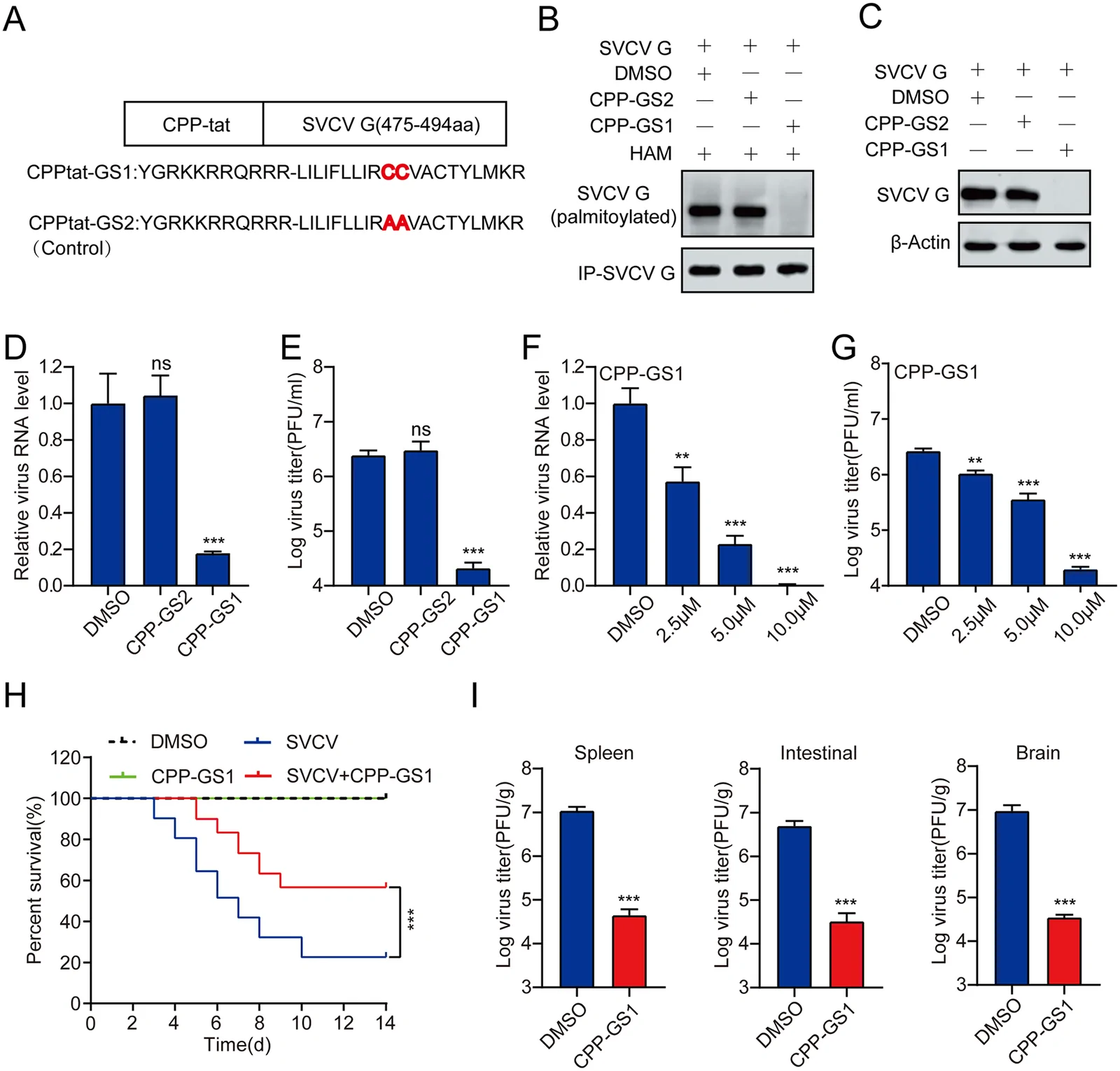
Targeting SVCV G palmitoylation with a peptide inhibitor. **(A)** Schematic of CPP-GS1 and CPP-GS2 peptides. The different residues are shown in red. CPPtat, CPP from HIV-1 Tat protein. **(B)** ZF4 cells were transfected with SVCV G for 6 h and then were incubated of CPPtat-GS1 and CPPtat-GS2 for 18 **h.** IP-ABE detection of palmitoylation of SVCV G in ZF4 cells. **(C)** ZF4 cells were transfected with SVCV G for 6 h and then were incubated of CPPtat-GS1 and CPPtat-GS2 for 18 **h.** Then, the cells were analyzed for SVCV G expression. **(D-E)** ZF4 cells were infected with SVCV G for 6 h and then were incubated of CPPtat-GS1 and CPPtat-GS2 for 18 **h.** Cell samples and supernatant were collected 24 h after infection. The viral RNA abundance was detected using qRT-PCR. SVCV titers were measured by Viral plaque assay. **(F-G)** ZF4 cells were infected with SVCV for 6 h and then were incubated with different concentrations of CPPtat-GS1 for 18 **h.** Cell samples and supernatant were collected 24 h after infection. The viral RNA abundance was detected using qRT-PCR. SVCV titers were measured by Viral plaque assay. **(H)** Zebrafish, with an average body weight of 0.6 **g.** At 6 h post-infection with 10^4^ PFU SVCV, zebrafish were divided into distinct treatment groups (n = 30) and administered either DMSO or the CPP-GS1 peptide (50mg/kg). The survival rate of the zebrafish was subsequently assessed 14 days post-injection. Statistics of survival rate were calculated with the log-rank (Mantel-Cox). ***p < 0.001. **(I)** The virus titer of SVCV at 5 dpi from the spleen, intestine, and brain after CPP-GS1 administration (n = 3 zebrafish per group). Data was presented as mean ± SD from three independent experiments. Statistical analysis was performed using Student’s *t*-*t*est and one-way ANOVA between different groups and the asterisk (*) indicates significant differences between groups*p < 0.05, **p < 0.01, ***p < 0.001.

To further evaluate the therapeutic efficacy of CPP-GS1 on SVCV infection *in vivo*. Zebrafish were infected with SVCV, followed by treatment with either DMSO or the CPP-GS1 peptide. A significant reduction in the survival rate was observed in the CPP-GS1-treated zebrafish, compared to the DMSO-treated group (Fig 5H). Furthermore, treatment with CPP-GS1 markedly reduced viral load across multiple tissues (Fig 5I). To evaluate the applicability of this antiviral strategy for other *rhabdoviruses* prevalent in low-temperature environments, we rationally designed a similar peptide, CPP-GI1, targeting the palmitoylation site of the IHNV G protein, and CPP-GV1, targeting the palmitoylation site of the VHSV G protein. CPP-GI1 inhibits IHNV replication in a dose-dependent manner (S8A-S8B Fig). At the same time, CPP-GV1 inhibits VHSV replication in a dose-dependent manner (S8C-S8D Fig). Subsequently, rainbow trout was infected with IHNV/VHSV and then treated with either DMSO or the CPP-GI1/CPP-GV1 peptide. As anticipated, treatment with CPP-GI1/CPP-GV1 resulted in an increased survival rate among IHNV/VHSV-infected rainbow trout and significantly reduced viral load across various rainbow trout tissues (S8E-S8H Fig). These findings indicate that the peptide targeting the palmitoylation of viral G proteins possesses therapeutic potential against *rhabdoviruses* that are adapted to low temperatures.

## Discussion

Many viruses exhibit variant infectivity and pathogenicity at different temperatures as they co-evolve with their hosts and the environment. To understand the mechanisms underlying this phenomenon, multiple studies have focused on the regulation of host immunity in response to different temperatures. However, a single host species can be infected by viruses from the same viral family under different environmental temperatures. For instance, zebrafish show high susceptibility to SVCV, VHSV, and IHNV at low temperatures; conversely, they are permissive to MSRV and SCRV at high temperatures. This observation implies that viral adaptation to temperature is influenced not only by host factors, including immune response [29] and gut microbiomes [30], but also by other viral specific variables. In this study, we found that during SVCV infection at a low temperature, its G protein undergoes significant palmitoylation. This modification is mediated by the host’s fatty acid synthesis pathway, which is activated in response to low-temperature adaptation (Fig 6). Furthermore, both IHNV and VHSV, which also demonstrate high infectivity at low temperatures, contain palmitoylation sites, with the number of these sites correlating with their adaptive temperatures. In contrast, no palmitoylation was observed in the G protein of MSRV and SCRV, which are prevalent at high temperatures. Thus, our research indicates that palmitoylation of the rhabdovirus G protein represents a key evolutionary adaptation for thriving in low-temperature environments.

**Fig 6:**
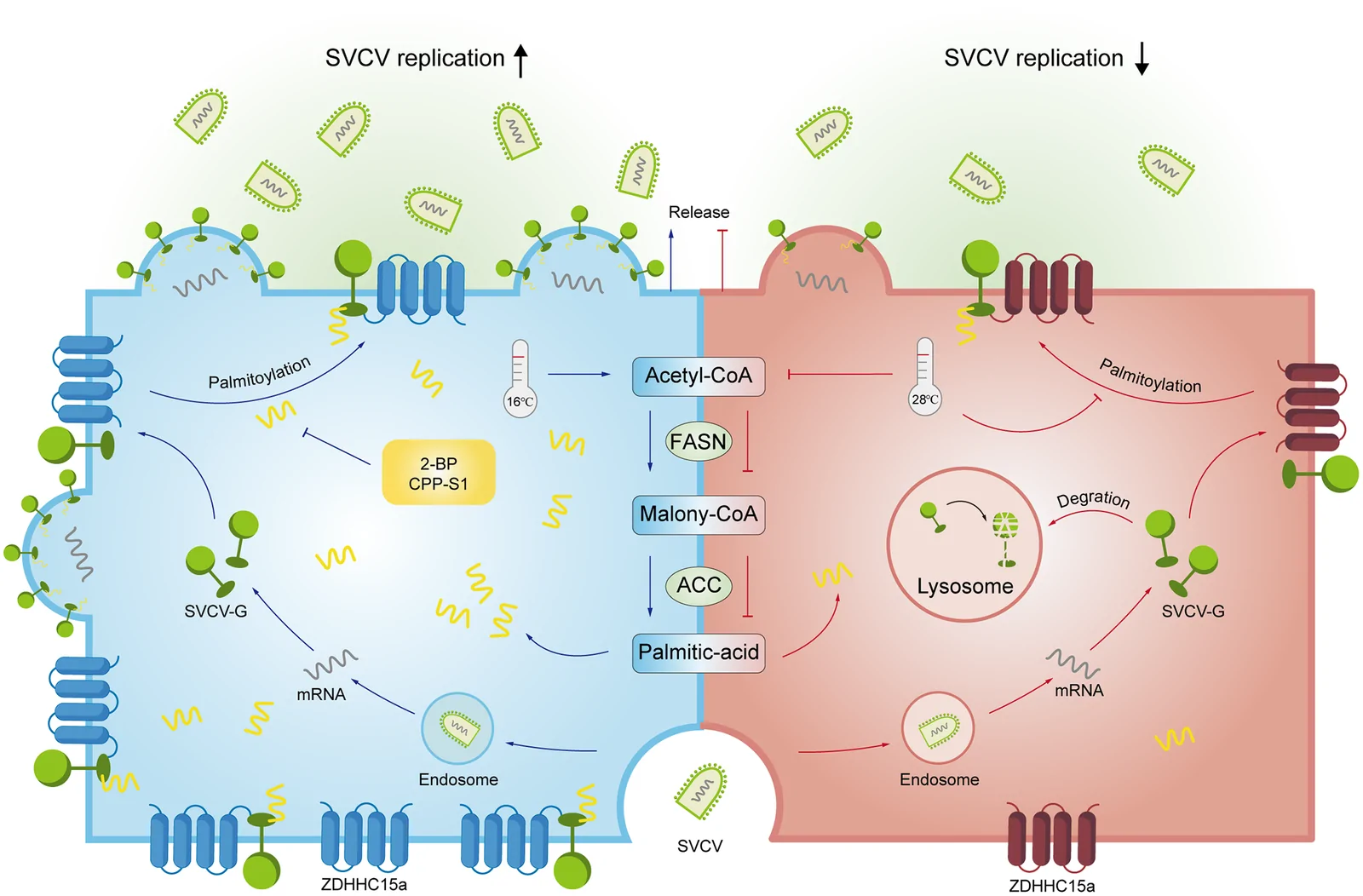
Schematic diagram displaying the overall process and mechanism of this study (Schematic diagram was created using adobe illustrator).

The interactions among viruses, hosts, and the environment are central to their co-evolution. Viruses frequently exploit host responses to environmental stress optimize replication and protein processing [31]. Environmental temperature significantly impacts fatty acid synthesis and metabolism in mammals [32,33]. Moreover, as poikilothermic vertebrates, teleost fish are unable to maintain a constant body temperature, which results in a more pronounced effect of temperature on the fatty acid metabolism of their hosts [34]. Through an integrated transcriptomic and metabolomic analysis, we found that bony fishes significantly upregulate fatty acid synthesis in the process of adapting to low temperatures. This finding is consistent with numerous studies indicating that poikilothermic animals regulate fatty acid synthesis to improve cell membrane fluidity as a response to low temperature conditions [35]. Metabolite-linked post-translational modifications (PTMs) are highly responsive to environmental or metabolic shifts, altering protein conformation, stability, localization, and interactions [36–38]. Unlike enzymatically regulated PTMs such as phosphorylation, the extent of metabolic PTMs often depends on precursor metabolite availability [39–41]. We observed that low temperatures activated palmitic acid synthesis, thereby providing a material basis for the low-temperature-induced palmitoylation modification of the G protein.

The adaptation of viral glycoprotein to host body temperature is also observed in respiratory viruses of zoonotic origin, most notably influenza viruses and SARS-CoV-2. The hemagglutinin (HA) glycoproteins of Influenza A (IAV) and B (IBV) viruses have evolved clear temperature preferences, which improves their fitness within hosts possessing different body temperatures [42,43]. SARS-CoV-2 demonstrates robust replication in the nasal cavity and upper respiratory tract; an adaptation achieved through precise tuning of its spike (S) protein to this specific anatomical environment [44,45]. It is particularly noteworthy that the HA proteins of both Influenza A and B viruses, and the S protein of SARS-CoV-2, are subject to significant palmitoylation modifications [26,46–48]. Therefore, the association between glycoprotein palmitoylation and temperature adaptation in rhabdoviruses may offer a viable framework for understanding the evolutionary temperature adaptation of influenza and novel coronaviruses. Nevertheless, it remains to be verified whether glycoprotein palmitoylation is similarly correlated with the temperature adaptation of these respiratory viruses.

Notably, several studies have shown that the L protein of vesicular stomatitis virus (VSV), a mammalian rhabdovirus, is involved in temperature sensitivity [49,50]. In the present study, while we could not directly prove the involvement of the SVCV L protein in the virus’s temperature sensitivity, prior research has established that the VHSV L protein contributes to the thermal limits of viral replication [51]. Our findings reveal a new role for the SVCV G protein in adapting to lower temperatures. Consequently, it is reliable that the G protein, in addition to the L protein, also be a factor in determining the replication temperature of rhabdoviruses.

Nevertheless, our study has its limitations. By targeting host metabolic remodeling, we identified a link between the palmitoylation of the rhabdovirus G protein and its temperature sensitivity. However, the absence of a SVCV reverse genetics system prevented us from further elucidating the role of the specific palmitoylation sites on the SVCV G protein in low-temperature adaptation and virulence. While previous research using the IHNV and VHSV reverse genetics system established the importance of its G protein in temperature sensitivity [4,52], the underlying mechanism and exact sites remained unclear. Our findings also confirm that the temperature-dependent mechanism of G protein palmitoylation is conserved in IHNV and VHSV, highlighting the critical role of rhabdovirus G proteins in the evolution of low temperature adaptation.

This conservation of palmitoylation sites in viral glycoproteins during evolution is consistent with findings in coronaviruses and alphaviruses, further illustrating the critical role of viral glycoprotein palmitoylation in viral replication [53,54].

In conclusion, our research clarifies the critical role of dynamic palmitoylation in the G protein of rhabdoviruses regarding their temperature sensitivity. This broadens the existing theoretical framework for understanding the pathogenic mechanisms of such viruses. Furthermore, this research holds significant importance for elucidating the mechanisms of virus-host-environment co-evaluation and provides critical targets for the development of anti-aquatic rhabdovirus strategies.

## Materials and methods

### Ethics statement

Every animal procedure conducted in this research strictly adhered to the guidelines established by China’s Ministry of Science and Technology in its publication on the ethical treatment of laboratory animals. Moreover, all experimental methodologies received proper validation and authorization from the Hubei Laboratory Animal Administration Committee, which granted approval under the reference number HZAUFI-2023–0130.

### Fish, cell lines and viruses

The AB zebrafish originated from the China Zebrafish Resource Center (CZRC), while rainbow trout were acquired from a Chongqing farm. Zebrafish embryo fibroblast-like (ZF4) cells (ATCC: CRL-2050) and zebrafish liver (ZFL) cells (ATCC: CRL-264) were cultured at 28 °C in Dulbecco’s Modified Eagle Medium F12 (DMEM/F12, Hyclone) enriched with 10% fetal bovine serum (FBS; Life Technologies). The propagation of SVCV, IHNV, and VHSV took place in ZF4 cells at 16 °C, harvested when approximately 80% virus-induced cytopathic effect was observed. The infected cultures were subjected to three cycles of freezing and thawing to release viral particles. The cell culture supernatant was then clarified by centrifugation at 10,000 × g for 10 min at 4 °C, and the resulting supernatant was collected and stored at −80 °C until use.

### Antibodies and reagents

The following reagents were acquired from ABclonal Technology: rabbit polyclonal antibodies targeting β-actin, ZDHHC15, and LAMP1, along with horseradish peroxidase-conjugated secondary antibodies from goat directed against rabbit and mouse IgG. Proteintech supplied the mouse monoclonal antibodies specific to GFP and Flag tags. Meanwhile, our laboratory maintained an in-house mouse monoclonal antibody against the SVCV G protein [55].

Firsocostat, Orlistat, Cycloheximide (CHX), MG132, 3-Methyladenine (3-MA), NH_4_Cl, and Bafilomycin A1 (Baf-A1) were procured from MedChemExpress, with 2-Bromopalmitic acid acquired from Sigma. We prepared stock solutions by dissolving all compounds in anhydrous DMSO.

CPP-GS1, CPP-GS2, CPP-GI1, and CPP-GV1 were all synthesized at TransGen Biotech. The concentration of the peptide used to treat ZF4 cells ranged from 2.5 µM to 10 µM. The concentration of the peptide injected into the fish was 50 mg/kg.

### Cell viability detection

Cell viability was assessed using an enhanced cell counting kit-8, following the manufacturer’s protocol to a tee. In a nutshell, ZF4 cells were plated in 96-well plates and treated with Firsocostat (2.5–20 nM), Orlistat (10–100 μM), 2-BP (10–100 µM), or DMSO as a control for 48 hours. After that, the CCK-8 solution was introduced to each well, and the plates were kept at 37 °C for one hour. Finally, the absorbance at 450 nm was read with a microplate reader from BioTek in Winooski, USA.

### Prediction of palmitoylation sites

Palmitoylation sites of G from SVCV, IHNV, VHSV and MSRV were predicted in CSS-palm 4.0 (http://gpspalm.biocuckoo.cn/download.php).

### Prediction of the 3D model

The 3D model in Fig 1H was generated using AlphaFold. Specifically, we retrieved the predicted structure of SVCV G from the AlphaFold Protein Structure Database (AFDB) using its UniProt accession number Q88415. The structure corresponds to the AlphaFold entry AF-0000000365761399, with a predicted Local Distance Difference Test (pLDDT) score of 73.95, indicating high confidence.

### Western blot

The protein samples underwent separation via 10% SDS-PAGE and were subsequently blotted onto 0.2 µm polyvinylidene fluoride membranes from Merck Millipore. Following this, the membranes were saturated with a blocking solution consisting of 2% fetal bovine serum in tris-buffered saline containing 0.05% Tween 20, left at room temperature for a couple of hours. They were then treated with the specified primary antibodies and incubated overnight at 4 °C. After washing with PBST five times, the membranes were exposed to HRP-conjugated secondary antibodies and allowed to incubate at room temperature for one hour. Chemiluminescencenoreactive bands were detected using ECL reagent on an Amersham Imager 600 chemiluminescence system by GE in the USA.

### qRT-PCR

Total RNA was isolated utilizing RNAiso Plus reagent, followed by cDNA synthesis employing the PrimeScript RT master mix kit. PCR amplification of the cDNA samples was performed with ChamQ universal SYBR qPCR master mix on the LightCycler480 II platform (Roche, Switzerland). Quantification of relative mRNA expression across samples was calculated via the 2^-ΔΔCT^ method, normalizing to Cc40S mRNA as an internal reference. Primer sequences are detailed in S1 Table.

### RNA interference

All small interfering RNAs (siRNAs), including the negative control (si-NC), were custom-designed and produced by GenePharma in Shanghai, China. Following the manufacturer’s guidelines, we introduced the specified siRNAs into the cells utilizing Lipofectamine RNAiMAX for transfection. These transfected cells were then used in the subsequent experimental procedures. For reference, the sequences of the designated siRNAs can be found in S1 Table.

### Confocal microscopy

ZF4 cells were plated in 20-mm culture dishes and subsequently transfected with the specified plasmids. Following a 24-hour overexpression period, cells underwent two PBS washes before being fixed with 4% PFA for one hour. When permeabilization was required, fixed cells were treated with Immunostaining Permeabilization Buffer containing Saponin (Beyotime Biotechnology; China). Cell nuclei were primarily labeled with DAPI, followed by three PBS washes, and then visualized using a confocal microscope (LSM800; Carl Zeiss AG, Germany).

### Palmitoylation analysis

The acyl-biotin exchange (ABE) protocol was implemented using well-established methodologies [56]. In brief, the prepared cells were combined with lysis buffer (comprising distilled water with 1% IGEPAL CA-630, 50 mM Tris–HCl at pH 7.5, 150 mM NaCl, 10% glycerol, 10 mM PMSF, and a complete protease inhibitor tablet) and maintained at 4 °C for 30 minutes. Subsequently, the cell lysate was transferred to a 1.5-milliliter microcentrifuge tube where it underwent sonication before being centrifuged at 13,000g for 10 minutes. The resulting supernatant was then pipetted into a fresh 1.5-ml tube, where palmitoylated proteins were processed using the IP-ABE Palmitoylation Kit (Aimsmass, catalog number AM10422) according to the manufacturer’s guidelines. The procedure commenced with blocking unmodified cysteine thiols via N-ethylmaleimide treatment, followed by specific cleavage of palmitoyl-cysteine bonds using hydroxylamine. Biotin was then introduced to bind with the newly freed sulfhydryl groups, and the biotin-tagged proteins were captured through incubation with streptavidin-conjugated magnetic beads (Beyotime, catalog number P2151). Post-incubation, the palmitoylated proteins were liberated through thermal denaturation and subsequently analyzed via western blot detection.

### Co-IP

The ZF4 cells were treated with the specified plasmids within 60-millimeter petri dishes for a duration of 36 hours. Upon completion, the cells were gathered and then subjected to lysis using Western blotting (WB/IP) lysis buffer, which had been enriched with a protease inhibitor blend. The cell lysate, or WCL, spun down at 12,000 grams for ten minutes to yield the supernatant. A tenth of the supernatant was set aside as a control sample. The leftover lysates were mixed with 20 microliters of either anti-Flag or anti-SVCV G magnetic beads and allowed to incubate overnight at 4 degrees Celsius. Subsequently, the beads were washed a total of six times and finally eluted using sodium dodecyl sulfate (SDS) loading buffer under high heat for ten minutes. The samples were then probed via Western blot analysis with the respective antibodies.

### CHX chase assay

ZF4 cells were transfected with SVCV G plasmid at 16 °C or 28 °C, or with SVCV G mutants. At 24 h after transfection, the medium was discarded, and the cells were treated with 1µg/mL chlorhexidine. Cells were harvested and lysed at the indicated time points (0, 1, 2, and 4 h). The samples were analyzed using Western blot.

### Virus titration assay

To determine the concentration of infectious viral particles in the culture medium from infected ZF4 cells, we performed a plaque assay. In this procedure, we systematically diluted the viral samples and applied them to cellular monolayers. Following a one-hour absorption period, we rinsed the cells with serum-free DMEM before transferring them to a maintenance medium comprising DMEM enriched with 3% fetal bovine serum and 1.5% sodium carboxymethyl cellulose (obtained from Sigma-Aldrich). After allowing three days for incubation, we tallied the observable plaque formations and employed these figures to compute the viral titers.

### Lysosome isolation

ZF4 cells underwent transfection with the plasmid carrying the SVCV G gene. Six hours after transfection, the groups overexpressing SVCV G were either exposed to 50 µM 2-BP or left untreated, while all cells received NH_4_Cl (0.5 mM) for a 24-hour period. Following this, the cells were collected, rinsed once with ice-cold PBS, and the supernatant was carefully discarded. Next, lysosomes were extracted from these cells using the Minute Lysosome isolation kit as per the manufacturer’s protocol. Ultimately, the prepared samples were heated in SDS loading buffer at 95 °C for 10 minutes and then analyzed via immunoblotting with the specified antibodies.

### Identification of the palmitoylation site using LC-MS/MS

Proteins were separated by SDS–PAGE, and the gel bands of interest were excised from the gel, reduced with 5 mM DTT, and alkylated with 11 mM iodoacetamide. Then, 1 M hydroxylamine hydrochloride was added to the gel and incubated for 3 h to reduce palmitate, and the gel was alkylated again with 11 mM ^13^C iodoacetic acid, followed by in-gel digestion with sequencing-grade modified trypsin at 37°C overnight. The peptides were extracted twice with 0.1% trifluoroacetic acid in a 50% acetonitrile aqueous solution for 30 min and then dried in a SpeedVac. Peptides were redissolved in 20 μl of 0.1% trifluoroacetic acid, and 6 μl of extracted peptides were analyzed by a Thermo Scientific Q Exactive mass spectrometer.

For LC-MS/MS analysis, the peptides were separated by 85-min gradient elution at a flow rate of 0.30 µl/min with a Thermo-Dionex Ultimate 3000 HPLC system, which was directly interfaced with a Thermo Scientific Q Exactive mass spectrometer. The analytical column was a homemade fused silica capillary column (75 µm ID, 150 mm length; Upchurch, Oak Harbor, WA) packed with C-18 resin (300 Å, 5 µm; Varian, Lexington, MA). Mobile phase A consisted of 0.1% formic acid, and mobile phase B consisted of 80% acetonitrile and 0.1% formic acid. The Q Exactive mass spectrometer was operated in the data-dependent acquisition mode using Xcalibur 2.1.2 software, and there was a single full-scan mass spectrum in the orbitrap (300–1,800 m/z, 70,000 resolution) followed by 20 data-dependent LC-MS/MS scans at 27% normalized collision energy (HCD).

### Generation of *zdhhc15a*-null zebrafish

Employing CRISPR/Cas9 genome editing, we targeted the zdhhc15a gene in zebrafish. The single-guide RNA design was crafted through the online platform accessible at http://crispr.mit.edu. To generate the gRNA template, we used the primer pair: forward 5′-GTAATACGACTCACTATAGGACAACGGCAGCCCAAGCCGTT

TTAGAGCTAGAAATAGC-3′ and reverse 5′-AAAAGCACCGACTCGGTGCC-3′. Synthesis of the single-guide RNA was carried out with the TranscriptAid T7 high-yield transcription kit from Fermentas. Following injection, embryos were kept at 28.5°C for a day before genomic DNA extraction from 20–30 embryos using a heat-based lysis method in 50 mM NaOH at 94°C for 40 minutes, halted by adding 1 M Tris-HCl (pH 8). Positive embryos were reared to adulthood as F0 founders and crossed with wild-type (WT) zebrafish to yield the F1 generation. Initial genotyping of F1 fish involved a heteroduplex mobility assay, with sequencing used to verify the target sites. F1 carriers of mutations were again backcrossed to WT zebrafish to produce F2 offspring. To rule out off-target effects, heterozygous zebrafish underwent at least five rounds of backcrossing with WT individuals. Subsequently, adult zebrafish with heterozygous genotypes (+/−) were interbred, resulting in progeny comprising wild-type (+/+), heterozygous (+/−), and homozygous (−/−) mutants. The primers for mutant detection were forward 5′-TGGACATTGCCATTGATCCAG-3′ and reverse 5′-CCATGCATTCCTCCTTGAAGA-3′.

### Statistical analysis

All experiments were performed in triplicate, and each assay was independently repeated at least three times. Data are presented as mean ± standard error of the mean (SEM). Statistical analyses were conducted using GraphPad Prism 7.0 (GraphPad Software, San Diego, CA, USA). Differences between groups were evaluated using Student’s *t*-test or one-way ANOVA. Statistical significance was indicated as follows: *P < 0.05; **P < 0.01; ***P < 0.001 and not significant findings (ns, P > 0.05).

## Supporting information

S1 Fig (A) Zebrafish, with an average body weight of 0.6 g, were intraperitoneally injected with either 10 μl of SVCV (1 × 10^7^ PFU/ml) or DMEM/F12 medium at varying temperatures. The survival rate of the zebrafish was subsequently assessed 14 days post-injection. Statistics of survival rate were calculated with the log-rank (Mantel-Cox). ***p < 0.001. **(B-C)** Rainbow trout, with an average body weight of 10 g, were intraperitoneally injected with either 10 μl of IHNV (1 × 10^7^ PFU/ml), VHSV (1 × 10^7^ PFU/ml) or DMEM/F12 medium at varying temperatures. The survival rate of the rainbow trout was subsequently assessed 14 days post-injection. Statistics of survival rate were calculated with the log-rank (Mantel-Cox). ***p < 0.001. **(D-F)** The abundance of Viral RNA levels in ZF4 cells infected with SVCV (MOI = 10), IHNV (MOI = 10) and VHSV (MOI = 10) were analyzed at different temperatures (16˚C, 28˚C) using qRT-PCR. **(G-I)** The virus titer in the supernatant of ZF4 cells infected with SVCV (MOI = 10), IHNV (MOI = 10) and VHSV (MOI = 10) at different temperatures (16˚C, 28˚C) was determined through virus titration. Data was presented as mean ± SD from three independent experiments. Statistical analysis was performed using one-way ANOVA between different groups and the asterisk (*) indicates significant differences between groups*p < 0.05, **p < 0.01, ***p < 0.001.
(TIF)

S2 Fig (A) The transcriptomic data of all gene differential regulation in spleen tissue samples of zebrafish at different temperatures were analyzed by volcano plot analysis. **(B)** KEGG pathway enrichment analysis was conducted on the transcriptome data of zebrafish spleen tissues at varying temperatures. The results were visualized using scatter plots, with axes based on enrichment factors and adjusted P-values. The enrichment factor is defined as the ratio of differentially expressed genes (DEGs) to the total number of genes within a specific gene set. In the scatter plot, the size of each point corresponds to the number of DEGs, while the color gradient reflects the significance level indicated by the *p*-value. **(C)** Metabolomic sequencing of zebrafish tissues at different temperatures, and KEGG pathway analysis after excluding non-lipid components. **(D)** Metabolites with significant changes in zebrafish tissues at different temperatures after the removal of non-lipid metabolites. **(E)** Determination of fatty acid content in zebrafish tissues at different temperatures by gas chromatography. Data was presented as mean ± SD from three independent experiments. Statistical analysis was performed using one-way ANOVA between different groups and the asterisk (*) indicates significant differences between groups*p < 0.05, **p < 0.01, ***p < 0.001.
(TIF)

S3 Fig (A-C) Cytotoxicity of Firsocostat, Orlistat and 2-BP in ZF4 cells. **(D-I)** ZF4 cells treated with different doses of ACACA inhibitor (0.5 μM, 1 μM, 2 μM) and FASN inhibitor were infected with SVCV (MOI = 10) at 16 °C. Cell samples and supernatants were collected 24 h after infection. (A and D) The viral RNA abundance was detected using qRT-PCR. (B and E) The SVCV G protein levels were analyzed by Western blotting. (C and F) SVCV titers were measured by Viral plaque assay. **(J-L)** ZF4 cells treated with different doses of 2-BP were infected with SVCV (MOI = 10) at 16 °C. Cell samples and supernatants were collected 24 h after infection. The viral RNA abundance was detected using qRT-PCR. The SVCV G protein levels were analyzed by Western blotting. SVCV titers were measured by Viral plaque assay. **(M)** Effect of 2-BP (50μM) on SVCV entry by qRT-PCR in ZF4 cells. **(N)** Effect of 2-BP (50μM) on SVCV replication by qRT-PCR in ZF4 cells. Intracellular viral RNA was quantified from intracellular viral RNAs at given time points by qRT-PCR. Data was presented as mean ± SD from three independent experiments. Statistical analysis was performed using one-way ANOVA between different groups and the asterisk (*) indicates significant differences between groups*p < 0.05, **p < 0.01, ***p < 0.001.
(TIF)

S4 Fig (A) ZF4 cells were transfected with the plasmid encoding SVCV G and were then treated with Orlistat (25µM, 50 µM, and 100 µM) or DMSO for 24 h at 16 °C. The SVCV G protein levels were measured by Western blotting. **(B-E)** ZF4 cells were transfected with the plasmid encoding SVCV G. The cells were treated with DMSO or 50 µM 2-BP, and then co-incubated with DMSO, 3-MA (2 mM), MG132 (20 nM), NH_4_Cl (0.5 mM), or Baf-A1 (40 µM) for 24 h at 16°C. The cells were lysed and analyzed by Western blotting. The levels of SVCV G expression were quantified by densitometry and normalized to that of β-actin. **(F)** ZF4 cells were transfected with the plasmid encoding SVCV G at 16°C and 28°C. At 6 h post-transfection, the SVCV G-overexpressed groups were treated with NH_4_Cl (0.5 mM) for 18 h at 16°C and 28°C. The lysosomes were isolated from these cells and analyzed by Western blotting. **(G-H)** ZF4 cells were transfected with the plasmid encoding SVCV G, SVCV G(C484/485A). At 6 h post-transfection, the SVCV G-overexpressed groups were treated with or without 50 µM 2-BP, and all the cells were treated with NH_4_Cl (0.5 mM) for 18 h at 16°C. The lysosomes were isolated from these cells and analyzed by Western blotting. **(I-K)** Analysis of SVCV G-protein levels on the cell membrane using ImageJ. **(L)** ZF4 cells were co-transfected with SVCV G and SVCV M for 24 h at 16°C. The protein levels of SVCV-M and SVCV-G in the cell lysate and culture supernatants were measured by Western blotting. Data was presented as mean ± SD from three independent experiments. Statistical analysis was performed using one-way ANOVA between different groups and the asterisk (*) indicates significant differences between groups ***p < 0.001.
(TIF)

S5 Fig (A) Changes in the expression levels of each acyltransferase in spleen tissue at different temperatures. **(B)** The expression of *zdhhc15a* in the brain, liver, spleen, intestine of zebrafish from different temperature were quantified by qRT-PCR. **(C)** The expression of *zdhhc15a* in ZF4 and ZFL cells from different temperature were quantified by qRT-PCR. **(D)** The protein level of ZDHHC15a in ZF4 cells from different temperature were quantified by Western blotting. **(E)** ZF4 cells were co-transfected with the plasmids encoding SVCV G and ZDHHC15a-Flag, with Flag-tagged empty vector as control for 24 h. The supernatant of WCL was immunoprecipitated using anti-Flag magnetic beads magnetic beads. Then, the precipitated proteins were analyzed by Western blotting. **(F)** ZF4 cells were transfected with the plasmid encoding SVCV G-GFP for 24 h. The cells were visualized with specific primary and secondary antibodies. Cell nuclei were stained with DAPI. Scale bars, 10 µm. **(G)** Scheme of targeting sites and the sequence information in *zdhhc15a*^-/-^ zebrafish. **(H)** Western blotting was used to detect the expression of ZDHHC15a protein in the spleen, intestine and brain tissues of *zdhhc15a*^-/-^ zebrafish (n = 3). **(I)** Photographs of bleeding on the surface and ascites of infected zebrafish (n = 3). Data was presented as mean ± SD from three independent experiments. Statistical analysis was performed using Student’s *t*-test between different groups and the asterisk (*) indicates significant differences between groups*p < 0.05, **p < 0.01, ***p < 0.001.
(TIF)

S6 Fig (A-D) ZF4 cells treated with different doses of FASN inhibitor and 2-BP were infected with VHSV (MOI = 10) at 16 °C. Cell samples and supernatants were collected 24 h after infection. The viral RNA abundance was detected using qRT-PCR. VHSV titers were measured by Viral plaque assay. **(E-H)** ZF4 cells treated with different doses of FASN inhibitor and 2-BP were infected with IHNV (MOI = 10) at 16 °C. Cell samples and supernatants were collected 24 h after infection. The viral RNA abundance was detected using qRT-PCR. IHNV titers were measured by Viral plaque assay. **(I-J)** ZF4 cells were co-transfected with the plasmids encoding VHSVG/IHNV G and ZDHHC15a-Flag, with Flag-tagged empty vector as control for 24 h. The supernatant of WCL was immunoprecipitated using anti-Flag magnetic beads magnetic beads. Then, the precipitated proteins were analyzed by Western blotting. **(K-L)** ZF4 cells were transfected with si-ZDHHC15a or si-NC for 12 h and then were transfected with the plasmid encoding VHSV G/IHNV G for 24 h. Protein palmitoylation was examined by IP-ABE assay. The palmitoylated VHSV G and the immunoprecipitated VHSV G were blotted by HRP-streptavidin and His mAb, respectively. **(M-N)** ZF4 cells were transfected with si-ZDHHC15a or si-NC for 12 h and then infected with VHSV(MOI = 10) for 48 h. Cell samples and supernatant were collected 48 h after infection. The viral RNA abundance was detected using qRT-PCR. VHSV titers were measured by Viral plaque assay. **(O-P)** ZF4 cells were transfected with si-ZDHHC15a or si-NC for 12 h and then infected with IHNV(MOI = 10) for 48 h. Cell samples and supernatant were collected 48 h after infection. The viral RNA abundance was detected using qRT-PCR. IHNV titers were measured by Viral plaque assay. Data was presented as mean ± SD from three independent experiments. Statistical analysis was performed using one-way ANOVA between different groups and the asterisk (*) indicates significant differences between groups*p < 0.05, **p < 0.01, ***p < 0.001.
(TIF)

S7 Fig (A) Schematic of the fusion motif GFP-GS1, including GFP and SVCV G (468–509), and the GFP-GS1 C484/485A mutant, GFP-GS2. **(B)** ZF4 cells were transfected with GFP-GS1 or GFP-GS2 plasmid at 16 °C, respectively, and observed under confocal microscopy. Scale bars, 10 μm. **(C)** ZF4 cells were transfected with GFP-GS1 or GFP-GS2 plasmid for 24 h at 16 °C, respectively. IP-ABE detection of palmitoylation of GFP-GS1 and GFP-GS2 expressed in ZF4 cells. **(D)** ZF4 cells were co-transfected with GFP-GS1/GFP-GS2 and SVCV G plasmid for 24 h at 16 °C, respectively. IP-ABE detection of palmitoylation of GFP-GS1 and SVCV G expressed in ZF4 cells. **(E)** ZF4 cells were co-transfected with GFP-GS1/GFP-GS2 and SVCV G for 24 h at 16 °C. Then, the cells were analysed for SVCV G expression. **(E-G)** ZF4 cells were transfected with GFP-GS1 and GFP-GS2 for 24 h at 16 °C. This was followed by SVCV (MOI = 10) infection. Cell samples and supernatant were collected 24h after infection. The viral RNA abundance was detected using qRT-PCR. The SVCV G protein levels were analyzed by Western blotting. SVCV titers were measured by Viral plaque assay. **(H)** ZF4 cells were co-transfected with the plasmids GFP-GS1 and ZDHHC15a-Flag, with GFP-GS2 as control for 24 h. The supernatant of WCL was immunoprecipitated using anti-GFP magnetic beads magnetic beads. Then, the precipitated proteins were analyzed by Western blotting. **(I)** ZF4 cells were co-transfected with the plasmids encoding SVCV G and ZDHHC15a-Flag, with Flag-tagged empty vector as control for 24 h. Then were incubated of CPPtat-GS1 and CPPtat-GS2 for 12 h. The supernatant of WCL was immunoprecipitated using anti-Flag magnetic beads magnetic beads. Then, the precipitated proteins were analyzed by Western blotting. **(J)** The ZF4 cells were incubated with FAM-labeled CPP-GS1, Observe under a fluorescence microscope 12 h later. Data was presented as mean ± SD from three independent experiments. Statistical analysis was performed using Student’s *t*-test between different groups and the asterisk (*) indicates significant differences between groups*p < 0.05, **p < 0.01, ***p < 0.001.
(TIF)

S8 Fig (A-B) ZF4 cells were infected with IHNV for 24 h and then were incubated with different concentrations of CPPtat-GI1 for 24 h. Cell samples and supernatant were collected 48 h after infection. The viral RNA abundance was detected using qRT-PCR. IHNV titers were measured by Viral plaque assay. **(C-D)** ZF4 cells were infected with VHSV for 24 h and then were incubated with different concentrations of CPPtat-GV1 for 24 h. Cell samples and supernatant were collected 48 h after infection. The viral RNA abundance was detected using qRT-PCR. VHSV titers were measured by Viral plaque assay. **(E)** Rainbow trout, with an average body weight of 10 g. At 6 h post-infection with 10^4^ PFU IHNV, rainbow trout were divided into distinct treatment groups (n = 30) and administered either DMSO or the CPP-GI1 peptide (50mg/kg). The survival rate of the Rainbow trout was subsequently assessed 14 days post-injection. Statistics of survival rate were calculated with the log-rank (Mantel-Cox). ***p < 0.001. **(F)** The virus titer of IHNV at 5 dpi from the spleen, intestine, and brain after CPP-GI1 administration (n = 3 rainbow trout per group). **(G)** Rainbow trout, with an average body weight of 10 g. At 6 h post-infection with 10^4^ PFU VHSV, rainbow trout were divided into distinct treatment groups (n = 30) and administered either DMSO or the CPP-GV1 peptide (50mg/kg). The survival rate of the rainbow trout was subsequently assessed 14 days post-injection. Statistics of survival rate were calculated with the log-rank (Mantel-Cox). ***p < 0.001. **(H)** The virus titer of VHSV at 5 dpi from the spleen, intestine, and brain after CPP-GV1 administration (n = 3 zebrafish per group). Data was presented as mean ± SD from three independent experiments. Statistical analysis was performed using Student’s *t*-test between different groups and the asterisk (*) indicates significant differences between groups ***p < 0.001.
(TIF)

S1 Table Primers of this study. (DOCX)

S1 Data Mass spectrometry data. Palmitoylation LC-MS analysis on ZF4 cells infected with SVCV under different temperatures (16 °C and 28 °C).
(ZIP)

S1 File Original images for blots and gels. (PDF)
