# Supplementary material for: Palmitoylation of glycoproteins under low temperature enhances SVCV replication
Source: PLoS Pathog. 2026 Sep 28;22(9):e1014654. doi: 10.1371/journal.ppat.1014654 (PMC13671642; doi:10.1371/journal.ppat.1014654)

Original images for blots and gels

**Fig1**

Fig1B

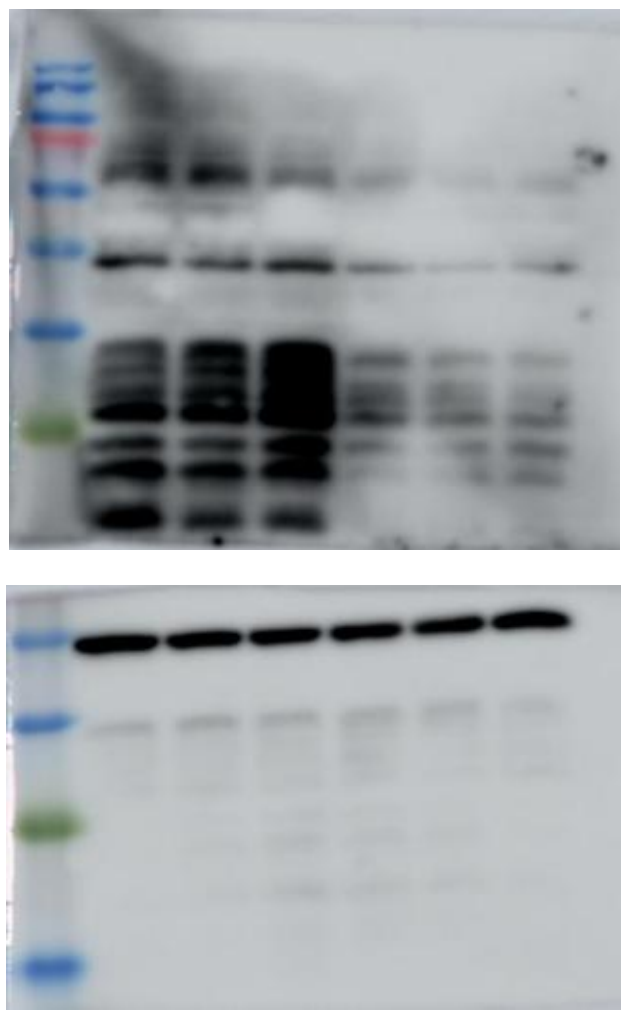

Fig1D

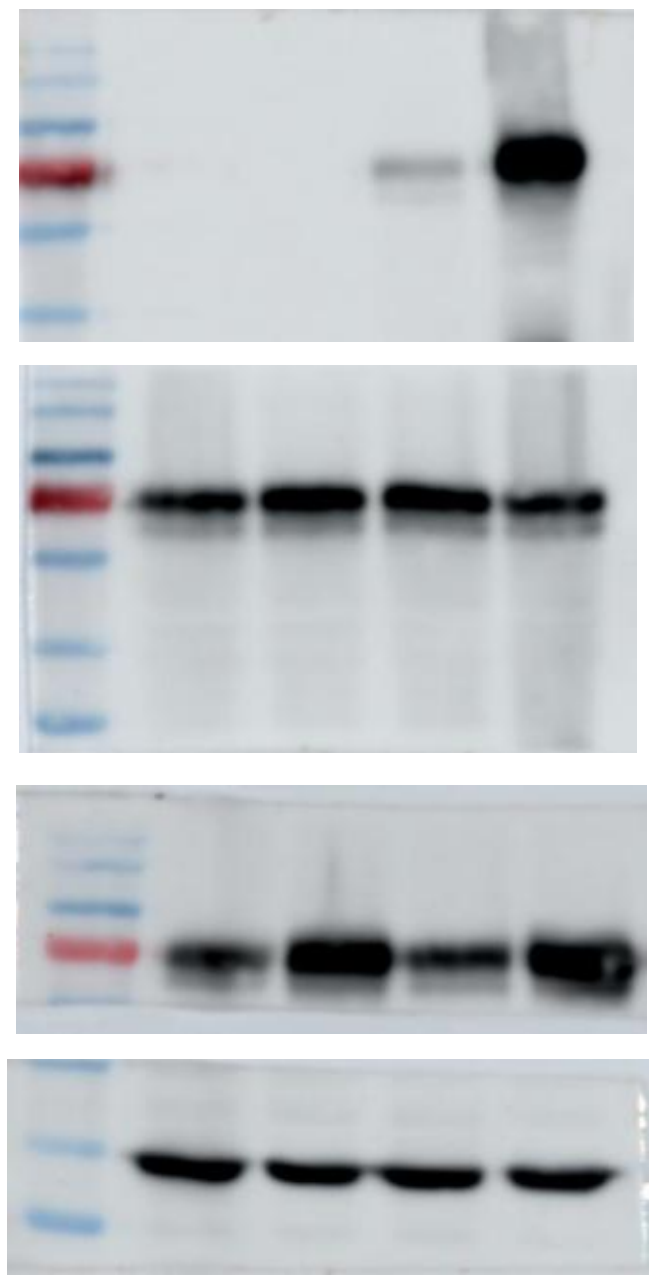

Fig1E

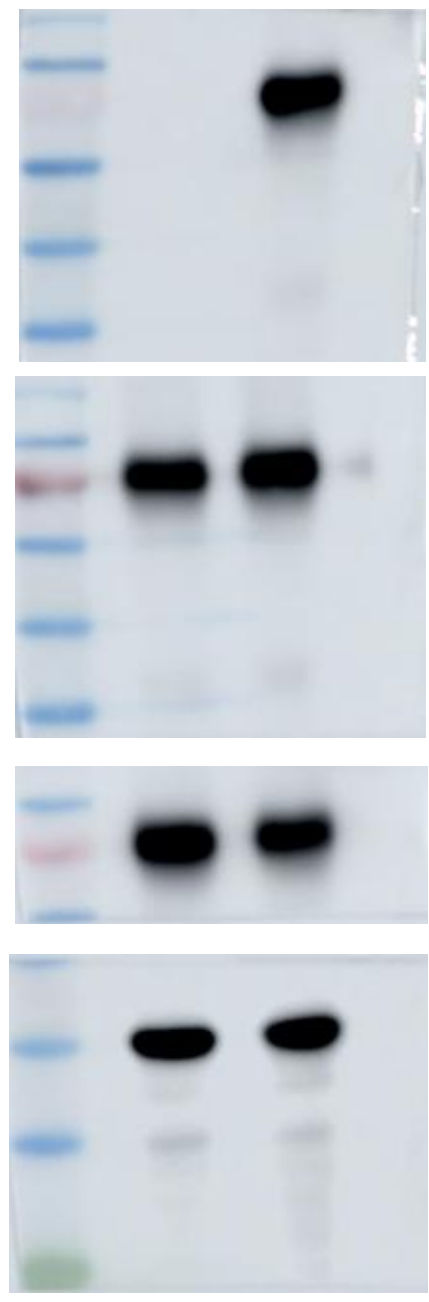

Fig1F

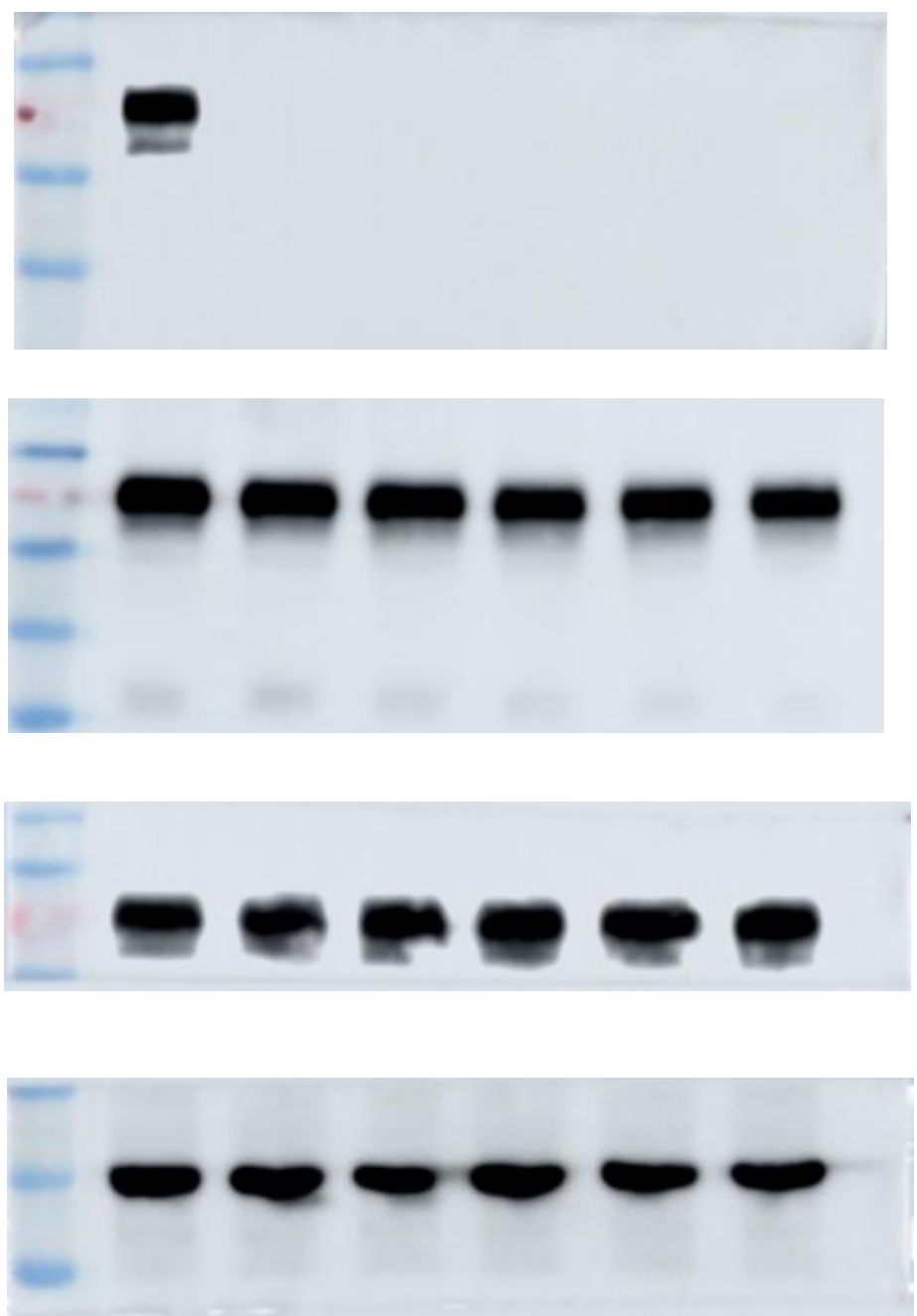

**Fig2**

Fig2A

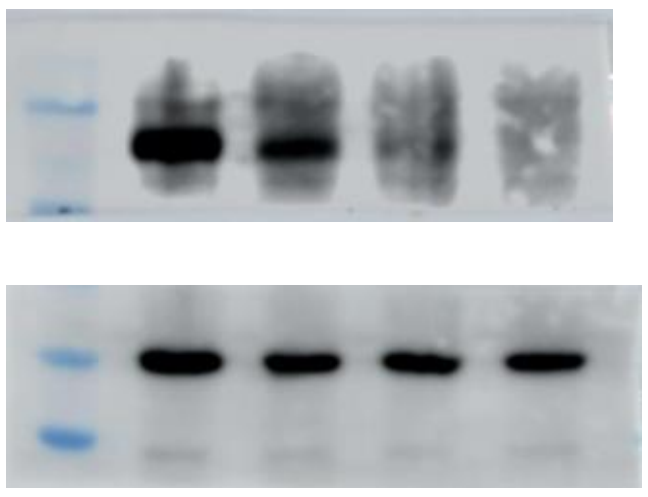

Fig2B

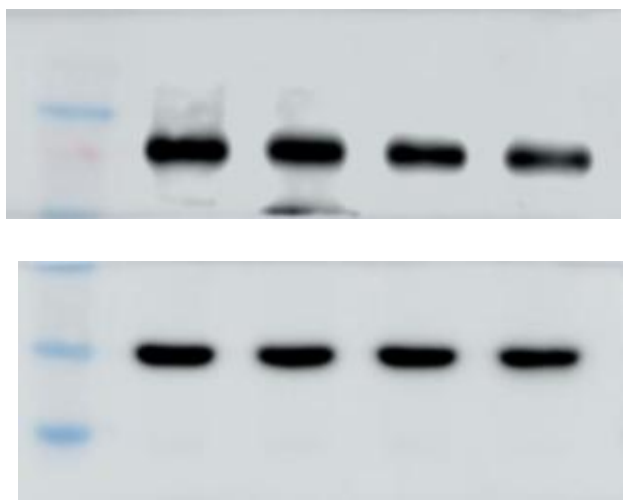

Fig2C

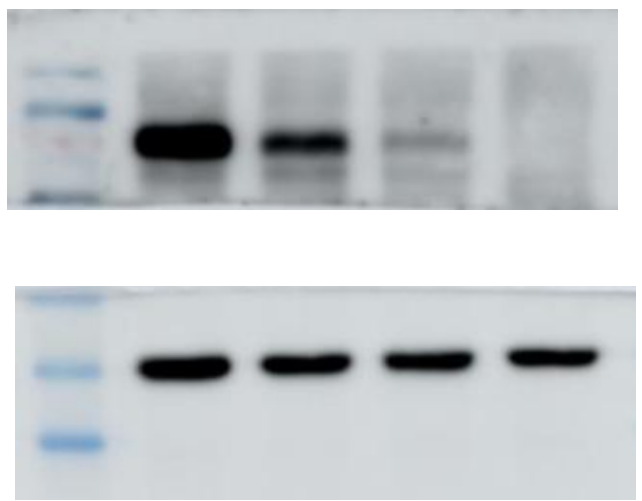

Fig2D

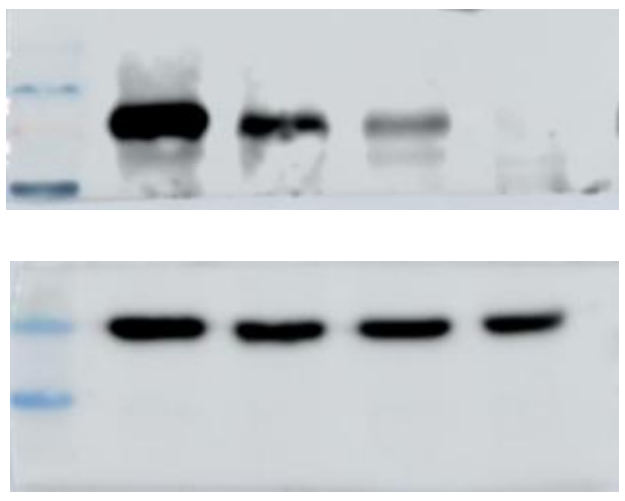

Fig2H

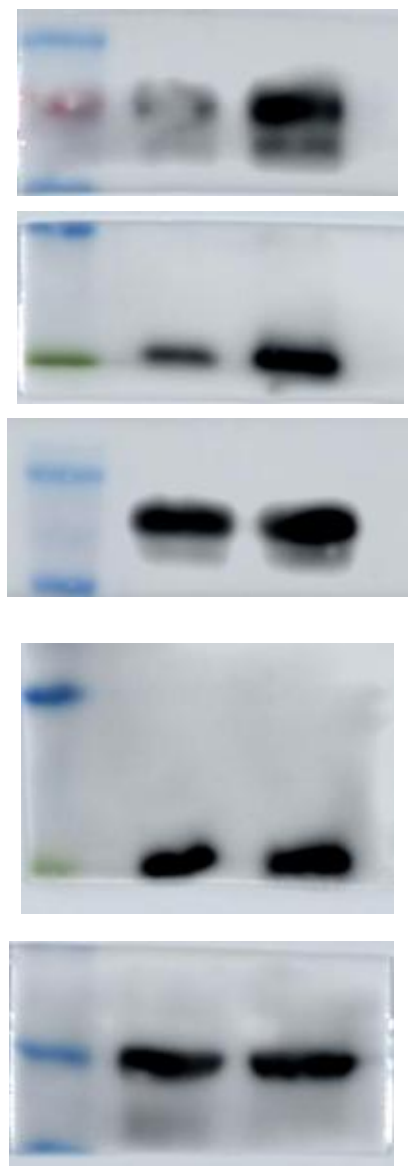

Fig2I

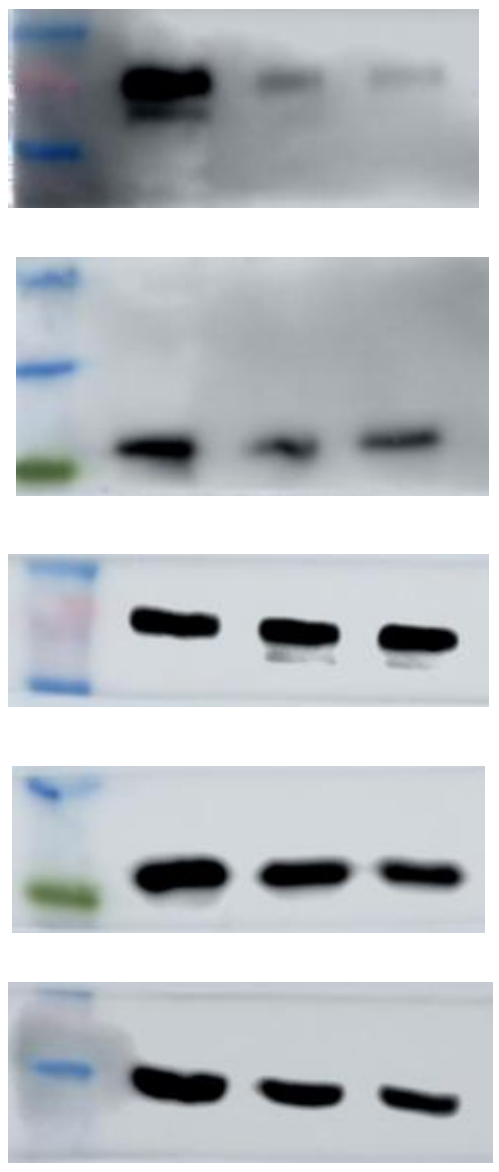

**Fig3**

Fig3B

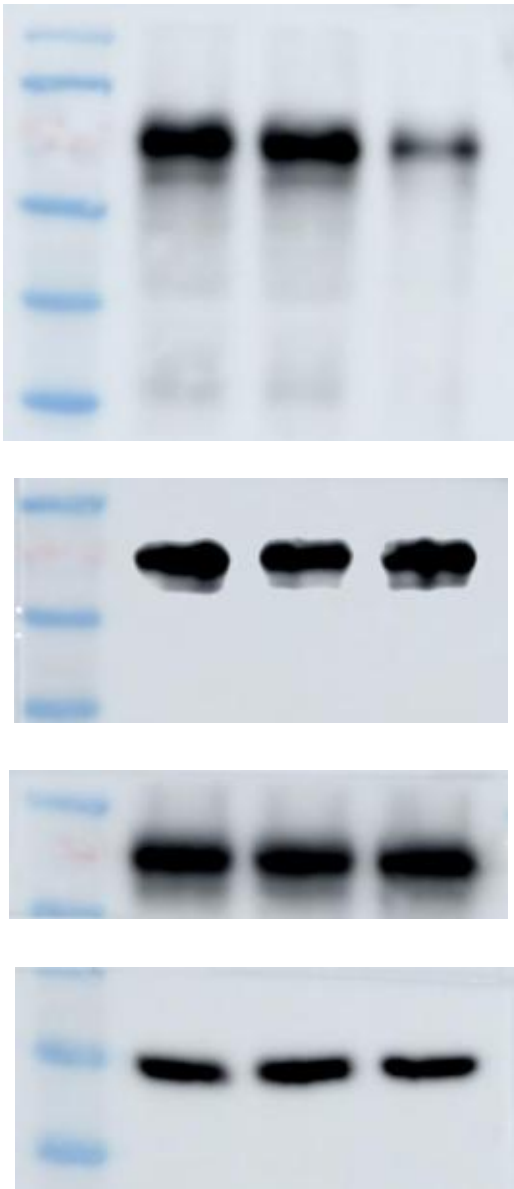

Fig3C

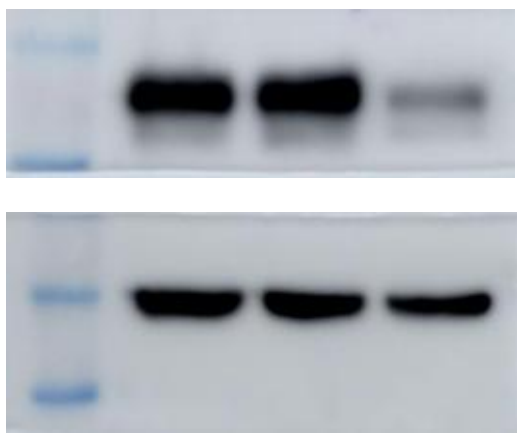

Fig3D

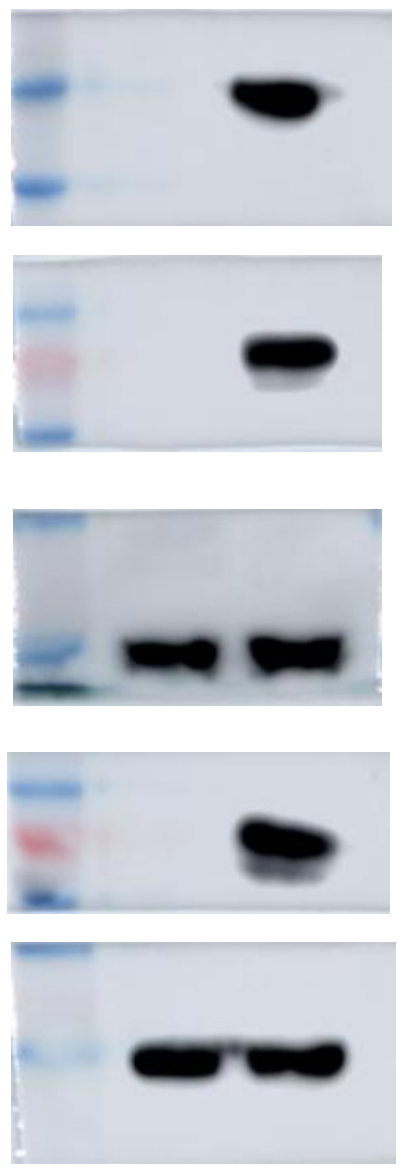

**Fig4**

Fig4B

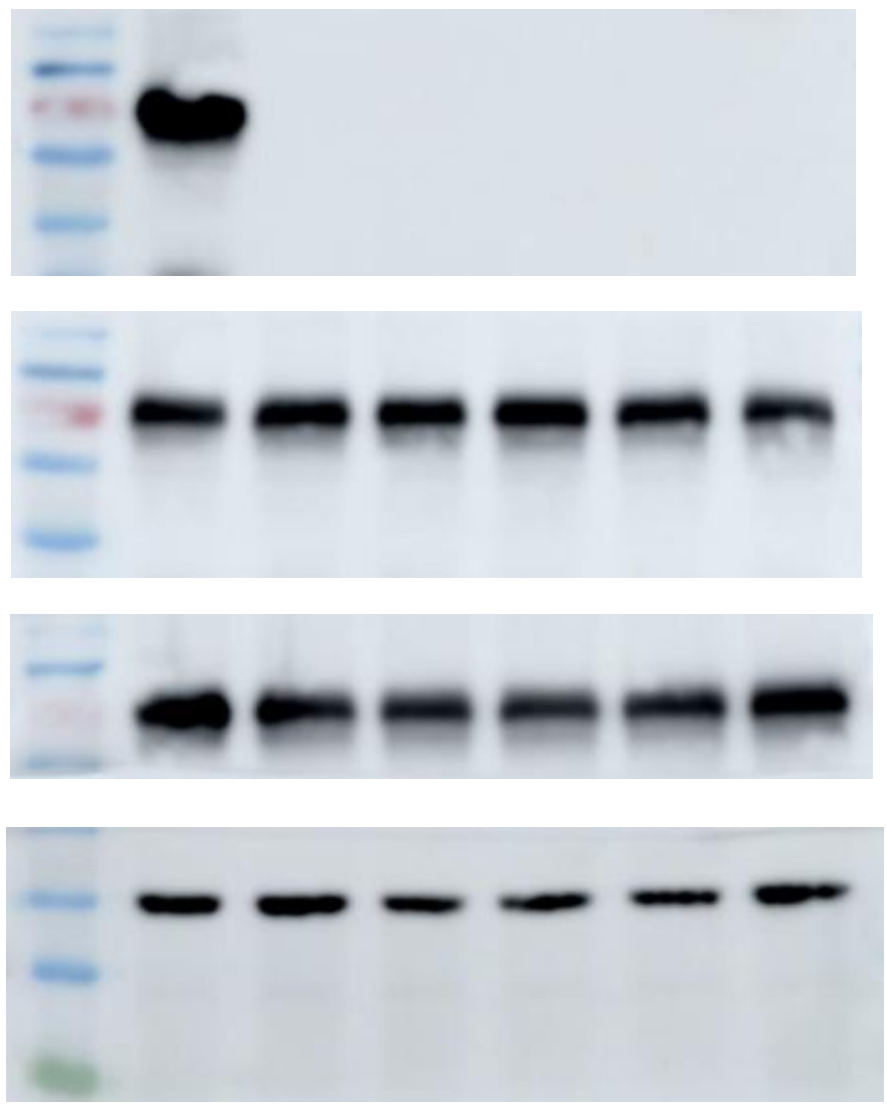

Fig4C

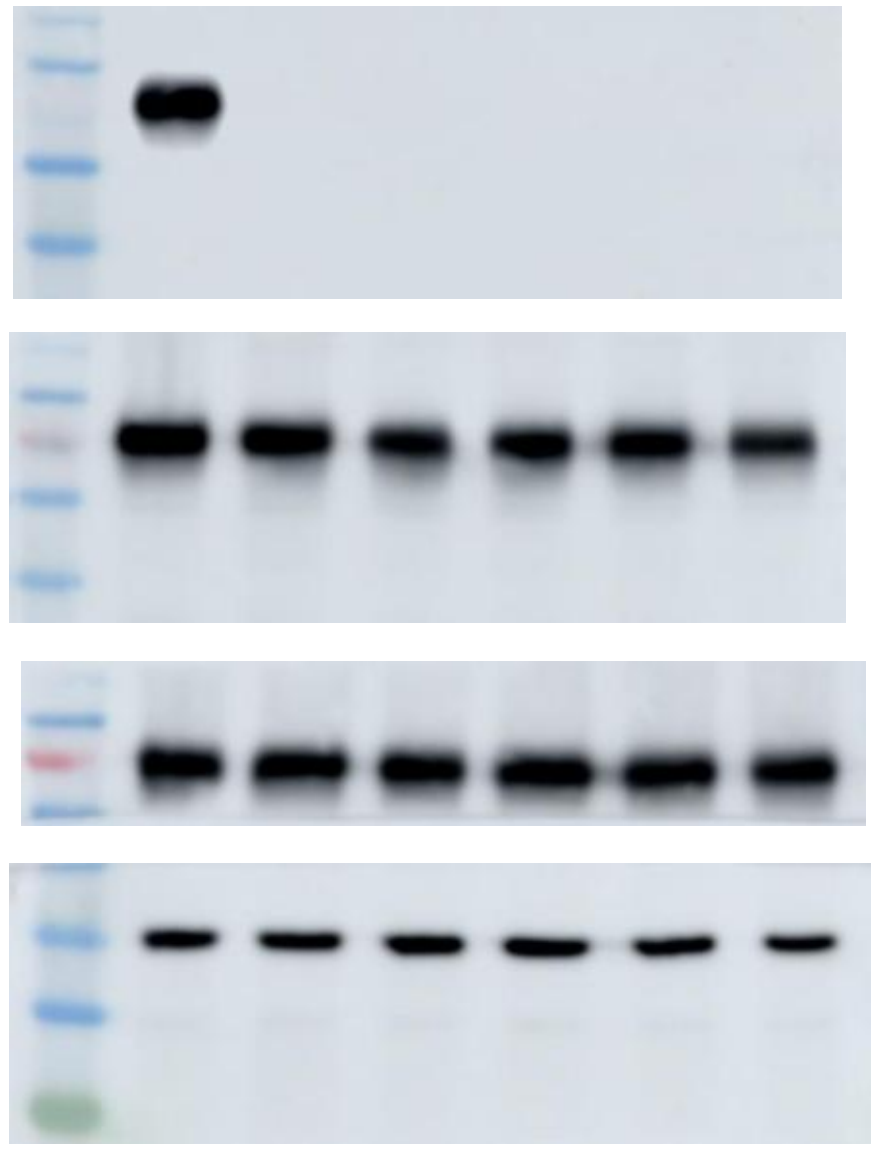

Fig4D

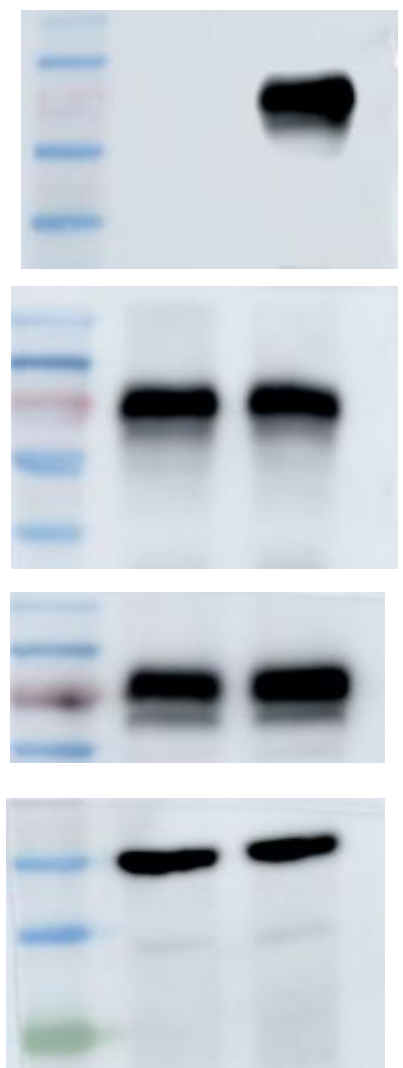

Fig4E

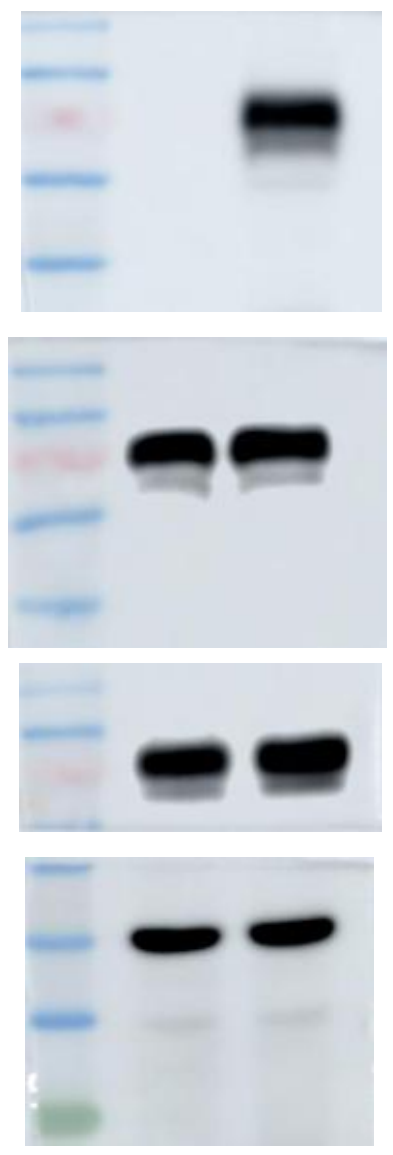

Fig4F

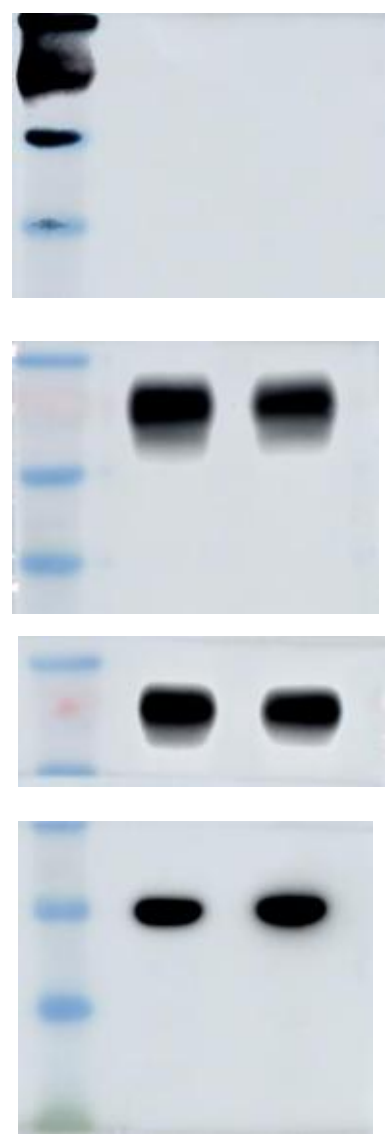

Fig4G

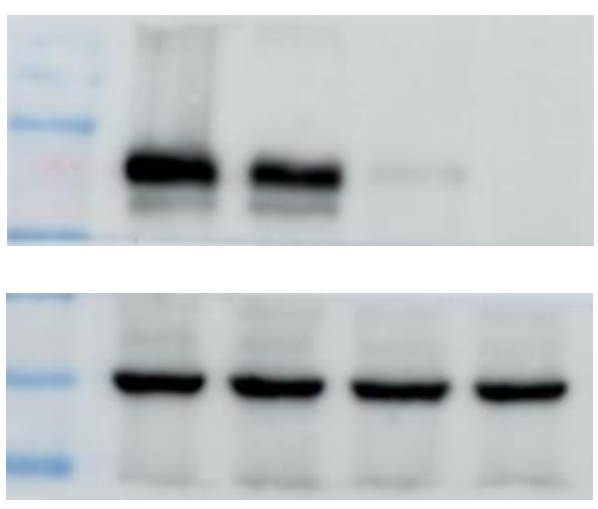

Fig4H

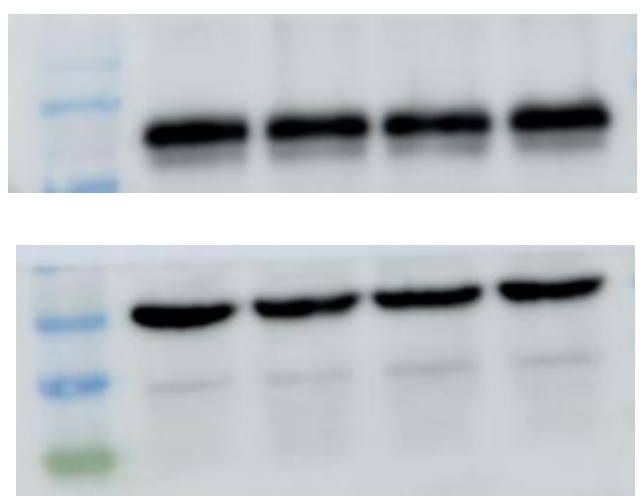

Fig4I

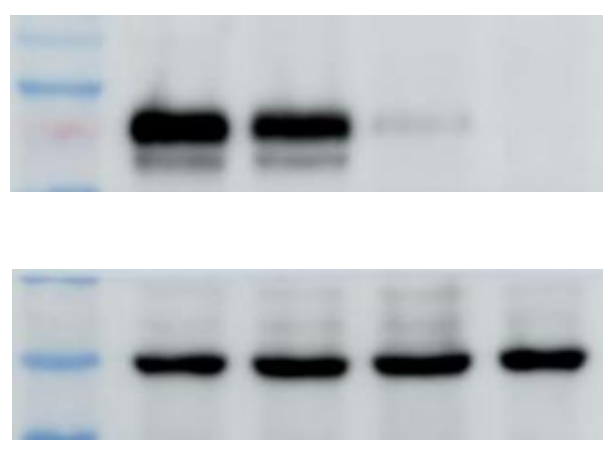

Fig4J

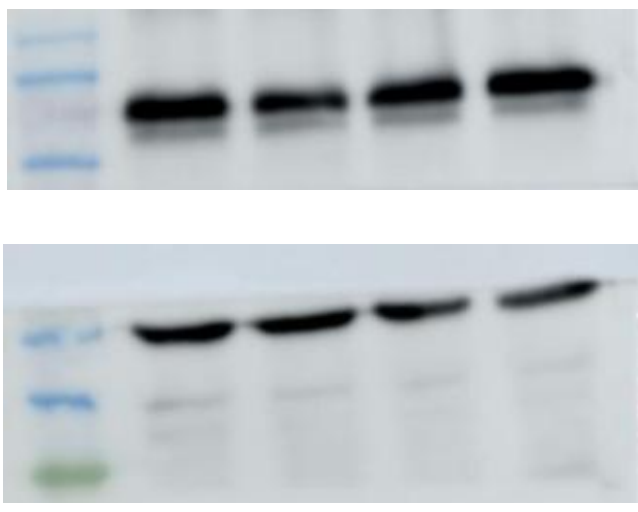

Fig4K

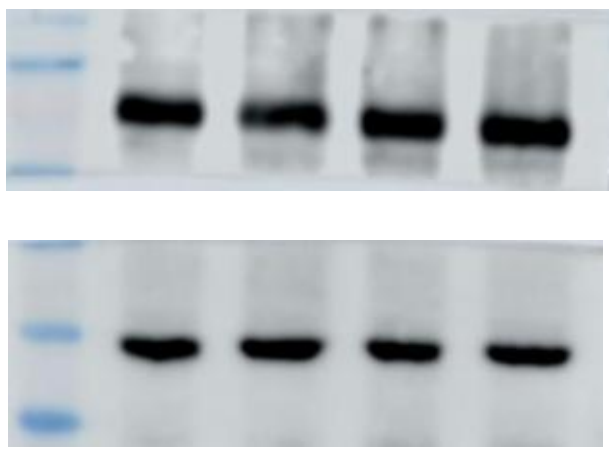

Fig4L

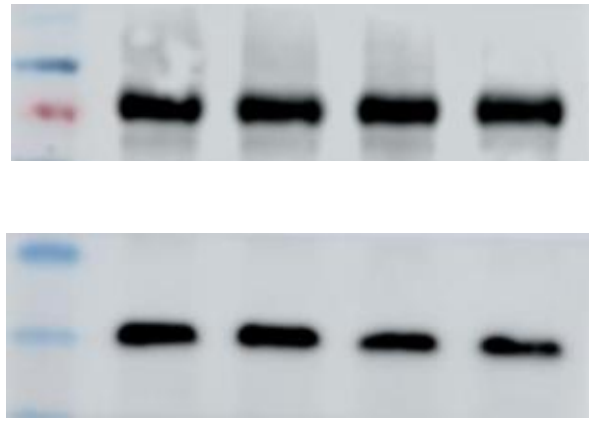

Fig4M

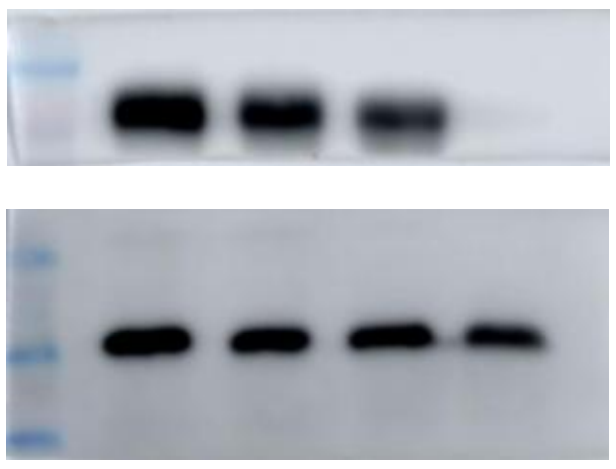

Fig4N

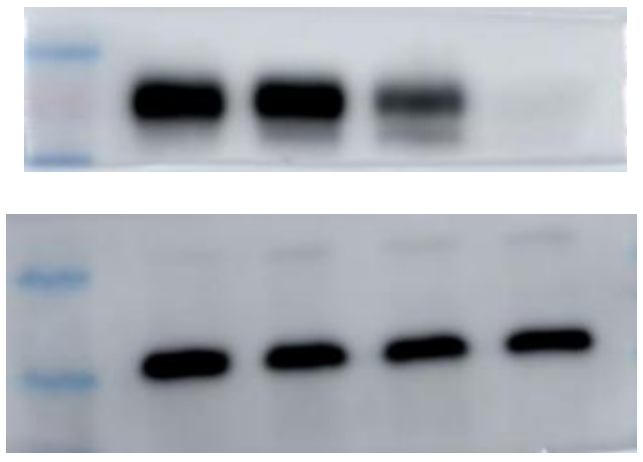

Fig4O

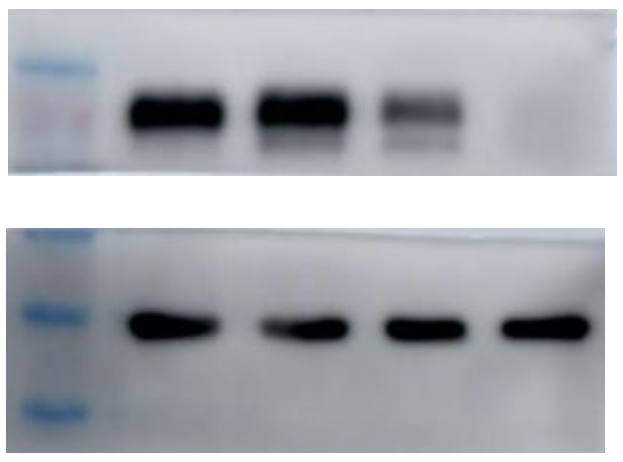

Fig4P

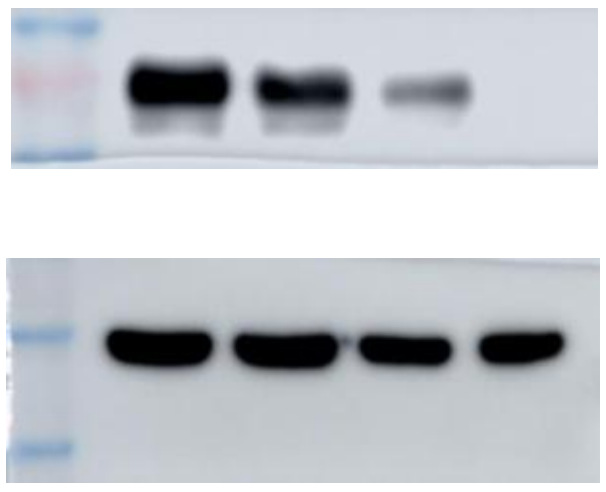

**Fig5**

Fig5B

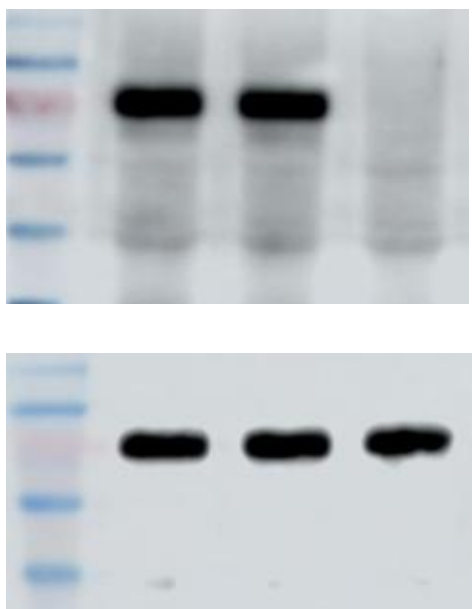

Fig5C

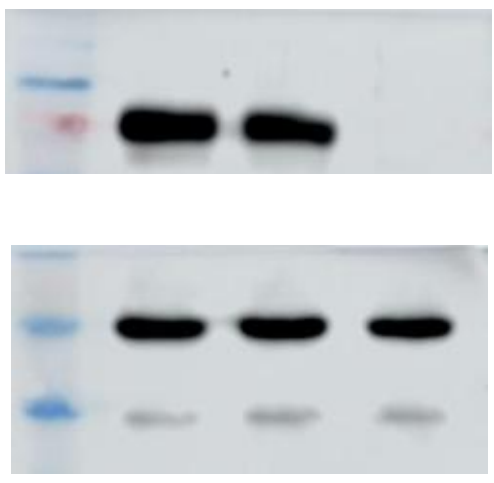

**FigS3**

FigS3E

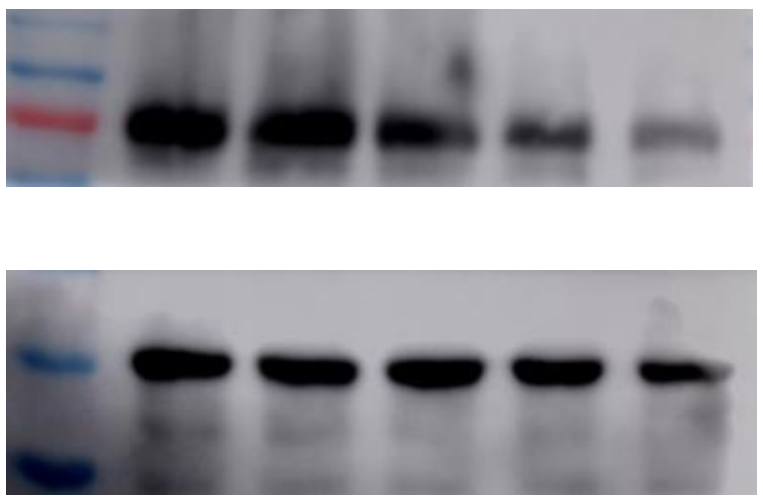

FigS3H

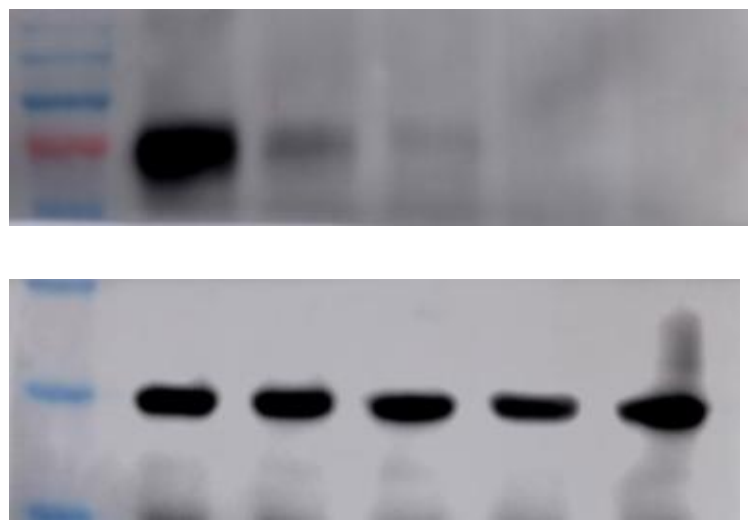

Fig3K

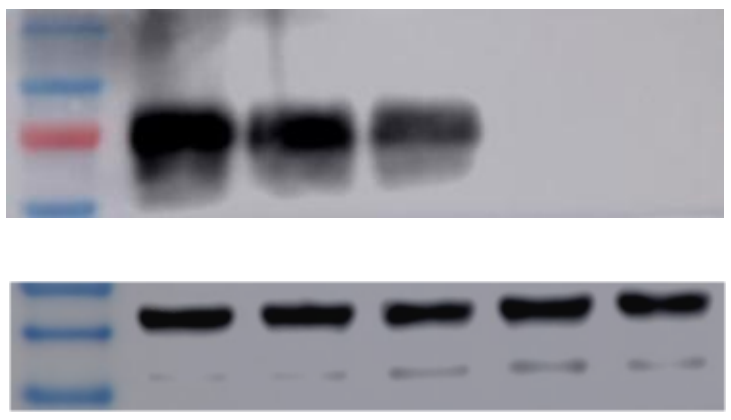

**FigS4**

FigS4A

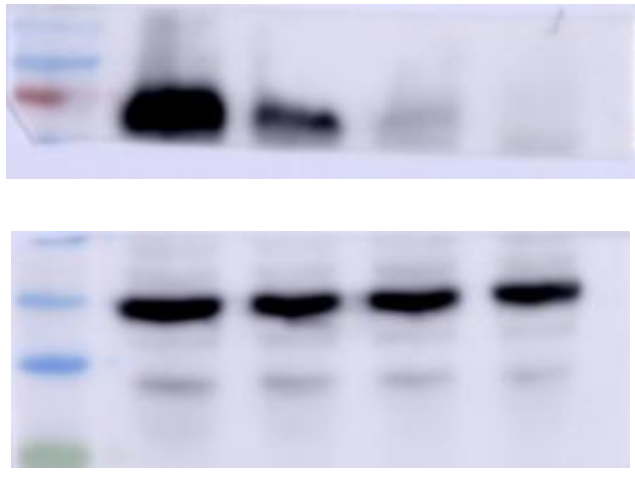

FigS4B

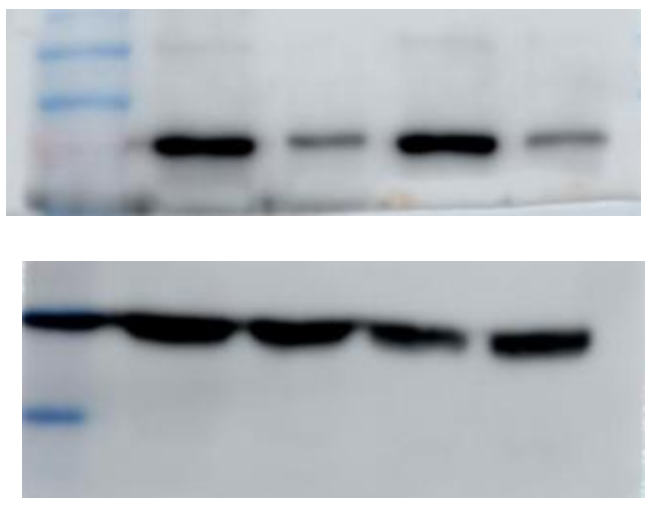

FigS4C

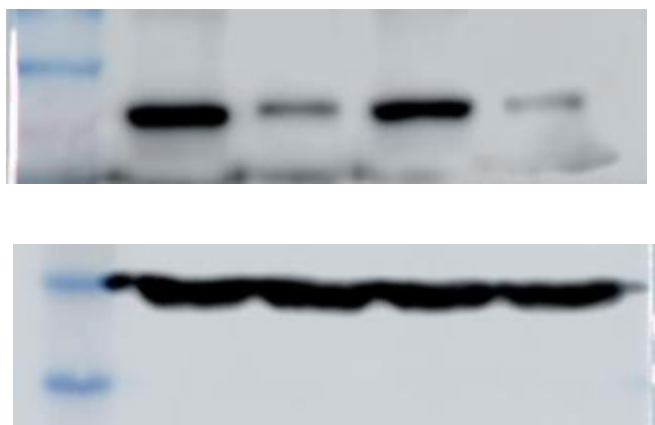

FigS4D

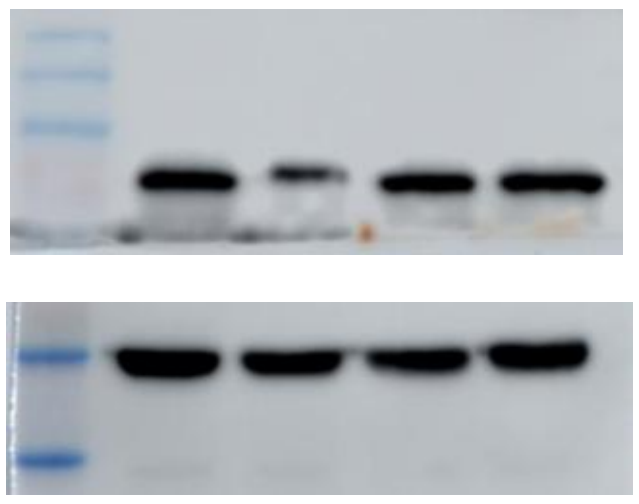

FigS4E

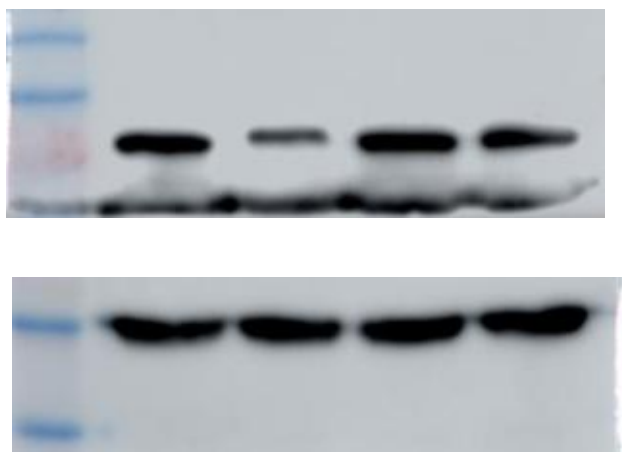

FigS4F

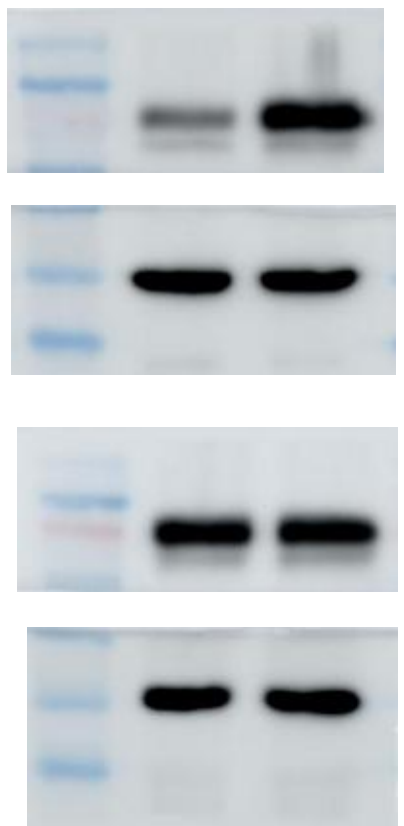

FigS4G

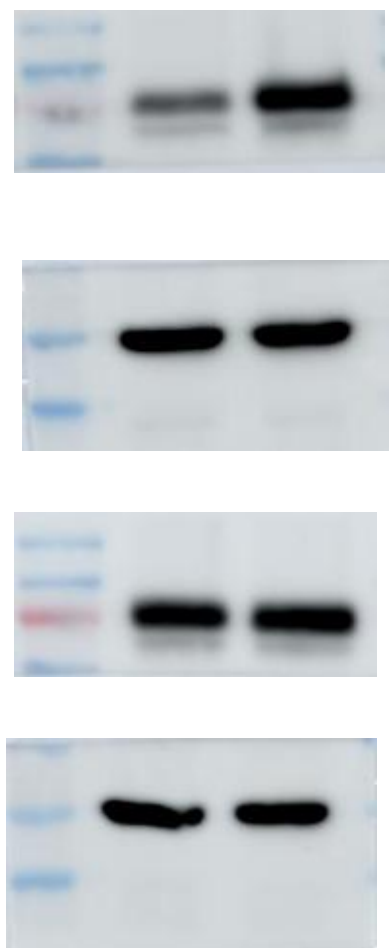

FigS4H

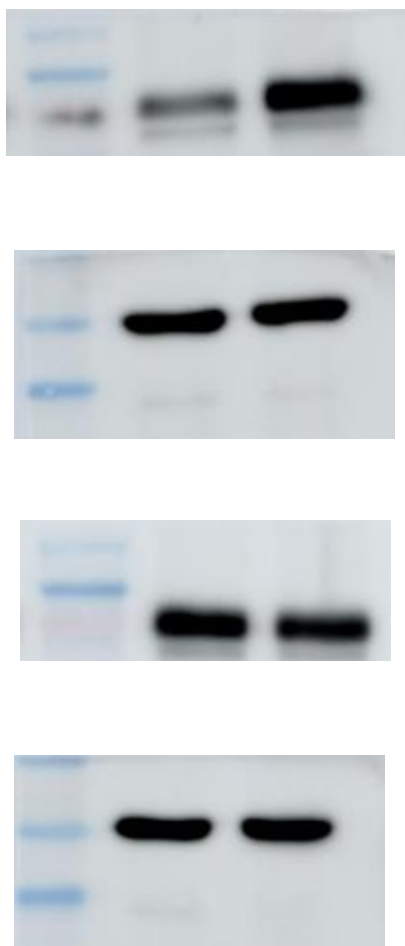

**FigS5**

FigS5D

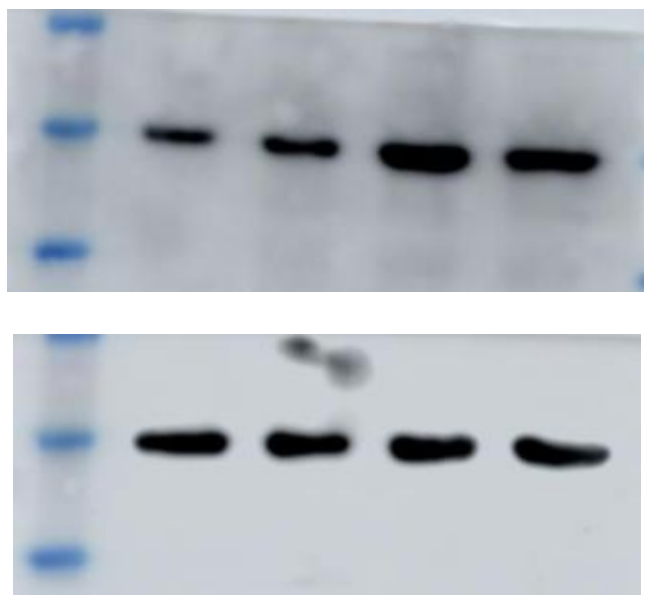

FigS5E

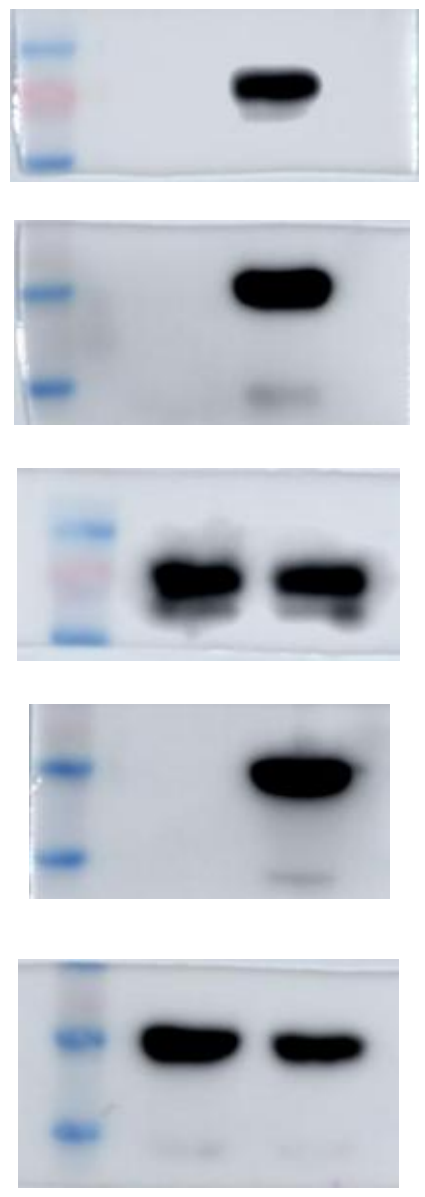

FigS5H

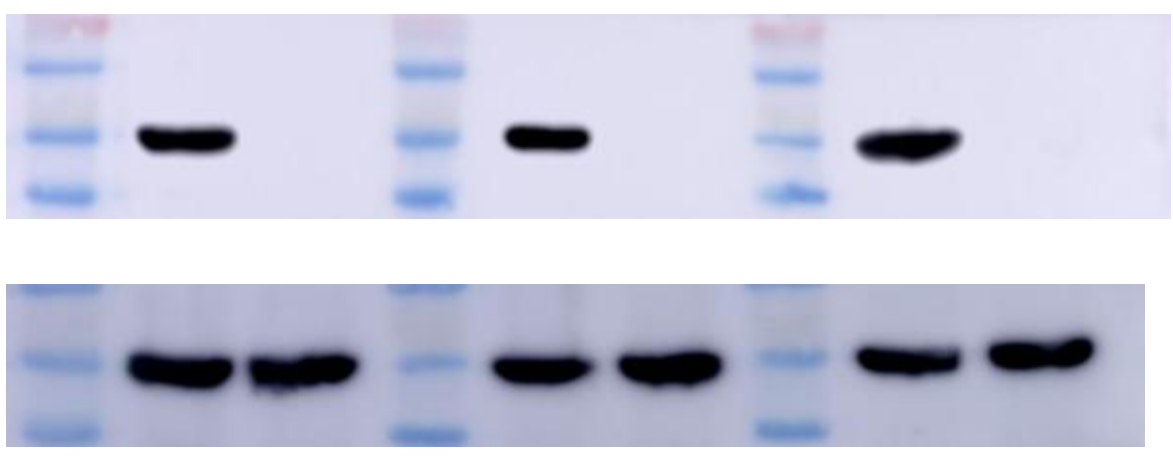

**FigS6**

FigS6I

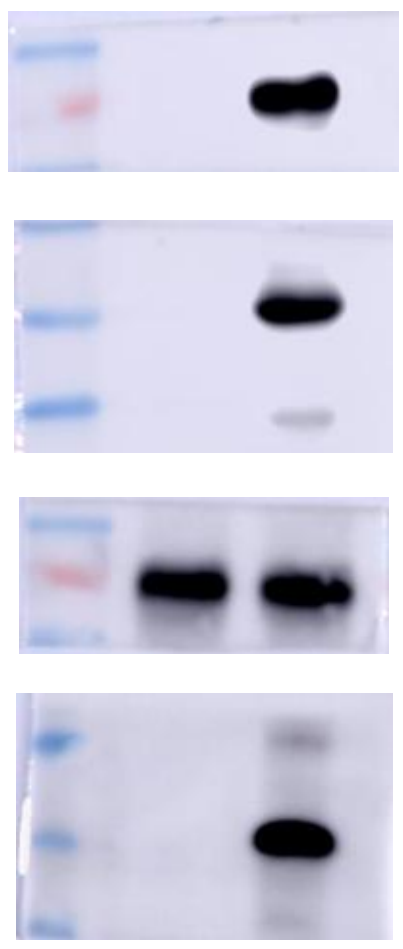

FigS6J

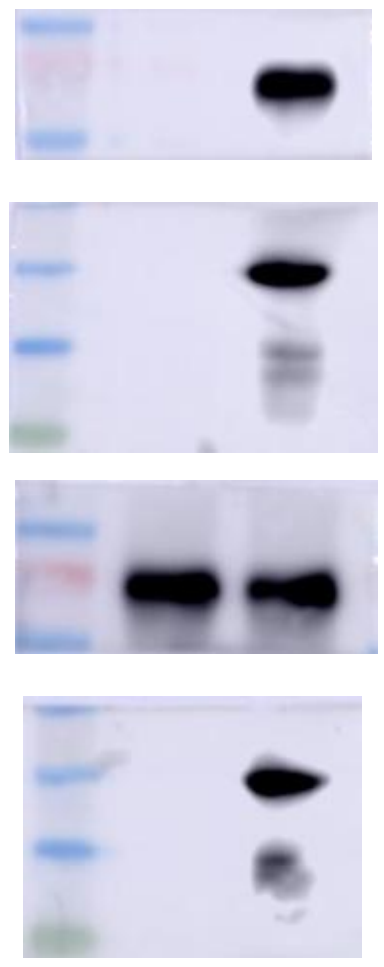

FigS6K

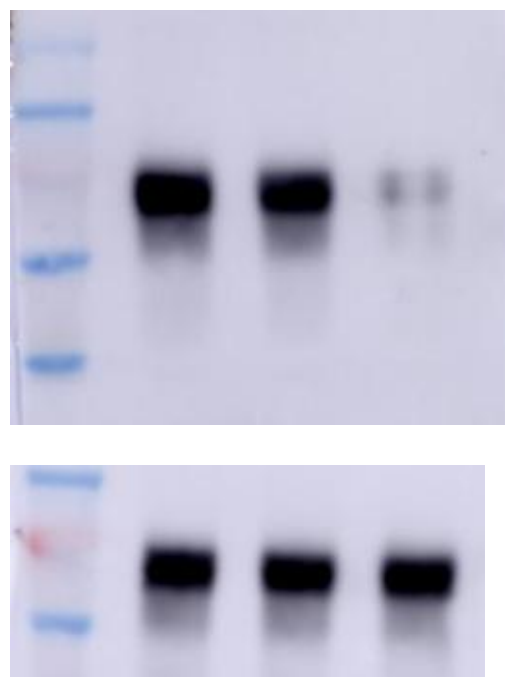

FigS6L

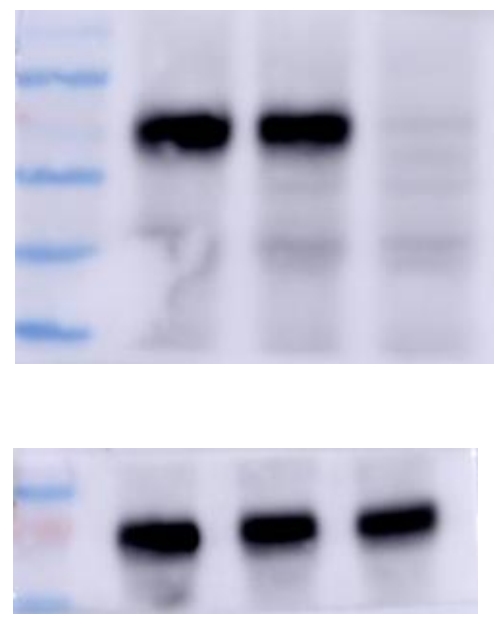

**FigS7**

FigS7B

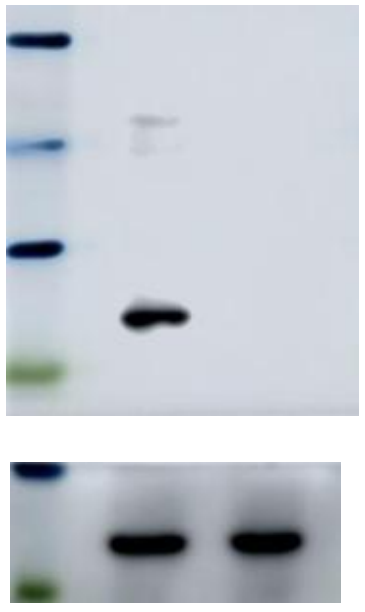

FigS7C

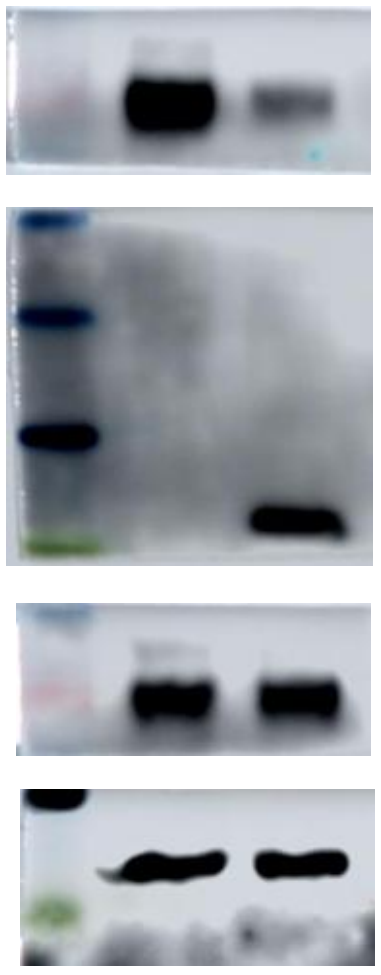

FigS7D

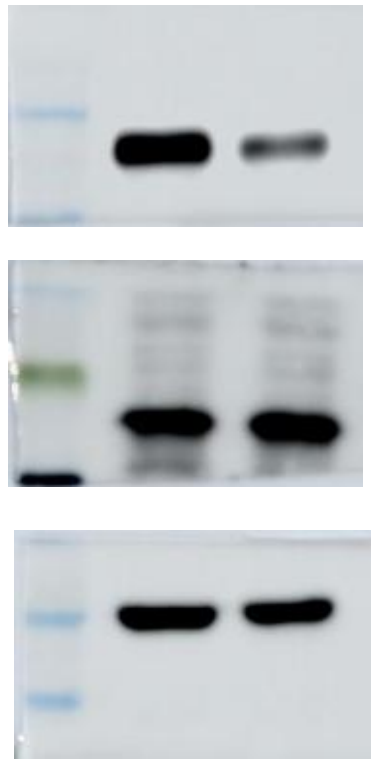

FigS7F

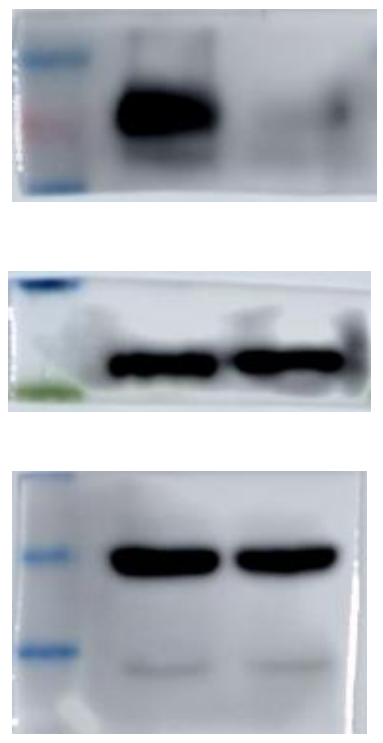

FigS7H

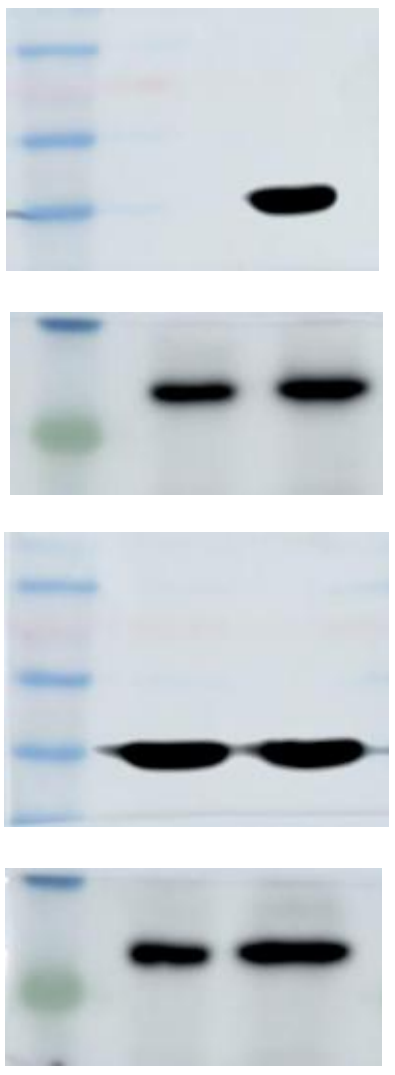

FigS7I

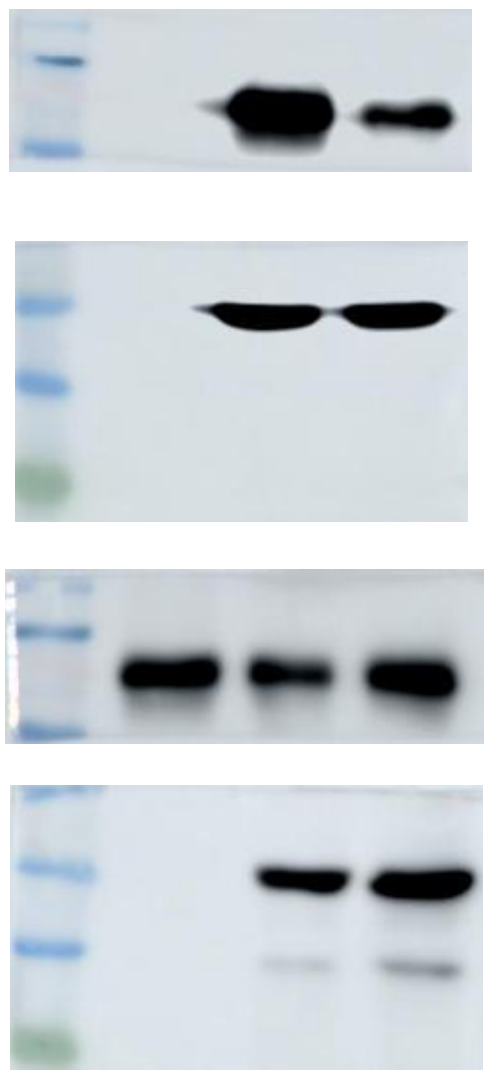

Supplement: Supplementary file 1 [file ppat.1014654.s011.pdf]
