# Supplementary material for: Palmitoylation of glycoproteins under low temperature enhances SVCV replication
Source: PLoS Pathog. 2026 Sep 28;22(9):e1014654. doi: 10.1371/journal.ppat.1014654 (PMC13671642; doi:10.1371/journal.ppat.1014654)
Supplement: Supplementary file 2 [file ppat.1014654.s009.docx]

**Table S1.**

Primers of this study.

| Application | Prime Name | Sequence (5′–3′) |
| --- | --- | --- |
| qRT-PCR  qRT-PCR  qRT-PCR  qRT-PCR | qCc40S-F  qCc40S-R  qSVCV G-F  qSVCV G-R | CCGTGGGTGACATCGTTACA  TCAGGACATTGAACCTCACTGTCT  CGACCTGGATTAGACTTG  AATGTTCCGTTTCTCACT |
| qRT-PCR | qIHNV G-F | TGTGACTCCATACCTCCTA |
| qRT-PCR | qIHNV G-R | CGTTCCCATTTCGTG |
| qRT-PCR  qRT-PCR  qRT-PCR  qRT-PCR  qRT-PCR  qRT-PCR  qRT-PCR  qRT-PCR  Plasmid construction  Plasmid construction  Plasmid construction  Plasmid construction  Plasmid construction  Plasmid construction  Plasmid construction  Plasmid construction  Plasmid construction  Plasmid construction  Plasmid construction  Plasmid construction  Plasmid construction  Plasmid construction  siRNA  siRNA | qVHSV G-F  qVHSV G-R  qACACA-F  qACACA-R  qFASN-F  qFASN-R  qZDHHC15a-F  qZDHHC15a-R  SVCV G-His-F  SVCV G-His-R  SVCV G(C/A)-His-F  SVCV G(C/A)-His-R  VHSV G-His-F  VHSV G-His-R  VHSV G(C/A)-His-F  VHSV G(C/A)-His-R  IHNV G-His-F  IHNV G-His-R  IHNV G(C/A)-His-F  IHNV G(C/A)-His-R  MSRV G-His-F  MSRV G-His-R  NC  NC | AGACGAGGCAAGCAA  TCACCGACCCAATAGA  ATCGCAGGTGAATCGTC  GCGTGGGTCATAAGGTG  GGACGAGTGCTTTACCC  ACGGACCCTGTCTTGG  GGCGTTTGATGTAGGTG  CAGTTCTTTGCGTGGC  CCCAAGCTTGCCACCATGTCTATCATCAGCTACATCGCAT  CCGCTCGAGAACTAAAGACCGCATTTCGTGT  CCCAAGCTTATGTCTATCATCAGCTACATCGCAT  CCGCTCGAGAACTAAAGACCGCATTTCGTGTGATTCTGTTGCAGGCCGTTTACTCCTCTTCATCAAATAAGTGCAAGCAACAGCGGCTCTGAT  CCCAAGCTTGCCACCATGGAATGGAACACTTTTTTCTTGG  CCGCTCGAGGATCATCTGACTTCTGGAGAACTGC  CCCAAGCTTATGGAATGGAACACTTTTTTCTTGG  CCGCTCGAGGATCATCTGACTTCTGGAGAACTGCTGCATCGGAATCCCGTAATTTGGAATGGGAGGGGACGCCTTGGCGGCGGCGGCGAGTAC  CCCAAGCTTGCCACCATGTACACCATGATCACCACTCCGC  CCGCTCGAGGGACCGGTTTGCCAGGTGATACATG  CCCAAGCTTATGTACACCATGATCACCACTCCGC  CCGCTCGAGGGACCGGTTTGCCAGGTGATACATGGGGATACTCTGCGGCGCGGAGGGAGTGGGAGGTCTCCCTGACCAGGCGGCGGCCGCGGC  CCCAAGCTTGCCACCATGAAATTGATCATTGCACCTACGC  CCGCTCGAGGGGAACAAATTGATACTGCTGCAAA  UUCUCCGAACGUGUCACGUTT  ACGUGACACGUUCGGAGAATT |
| siRNA  siRNA  siRNA  siRNA  siRNA  siRNA | SiZDHHC15a-1-F  siZDHHC15a-1-R  siZDHHC15a-2-F  siZDHHC15a-2-R  siZDHHC15a-3-F  siZDHHC15a-3-R | GUUUGACAGUUGUGUUGAATT  UUCAACACAACUGUCAAACTT  CGUGUGUGUUAAAGAUGGATT  UCCAUCUUUAACACACACGTT  GUGGAGUUUCAGUUCUCUATT  UAGAGAACUGAAACUCCACTT |
